# Accelerating ASEAN’s energy transition in the power sector through cross-border transmission and a net-zero 2050 view

**DOI:** 10.1016/j.isci.2024.111547

**Published:** 2024-12-06

**Authors:** Sheng Zhong, Lingyi Yang, Dimitri J. Papageorgiou, Bin Su, Tsan Sheng Ng, Saifudin Abubakar

**Affiliations:** 1 Energy Studies Institute, National University of Singapore, Singapore 119620, Singapore; 2 ExxonMobil Research and Engineering Company, Annandale, NJ 08801, USA; 3 Department of Industrial Systems Engineering and Management, National University of Singapore, Singapore 117576, Singapore; 4 ExxonMobil Asia Pacific Pte. Ltd., Singapore 098633, Singapore

**Keywords:** Energy engineering, Power Engineering, Energy sustainability, Energy management

## Abstract

Faced with energy transition objectives, the ten countries of the Association of Southeast Asian Nations (ASEAN) have technology options to decarbonize power sector. This study investigates the hypothetical decarbonization pathways for ASEAN’s power sector. Here, we present an integrated power system capacity expansion model for ASEAN over 2018–2050. The results identify different pathways by strategically pursuing renewable energy, carbon capture and sequestration, and cross-border transmission. Transmission, while accounting for a marginal share in total cost (up to 0.5%), can reduce cumulative system costs by 11.9% and help achieve net-zero emissions. Across scenarios, generation infrastructure will require a cumulative investment equivalent to 29.6%–44.6% of ASEAN’s 2018 GDP (Gross Domestic Product). The investment requirements for the expansion plan, however, are unevenly distributed across countries, especially with the ambition to achieve a carbon-neutral power sector. Country-specific investments in decarbonization are a consideration for the region.

## Highlights

• The study identifies different decarbonization pathways for ASEAN’s power sector
• Achieving net-zero requires an accelerated transition toward renewable energy
• Cross-border transmission can significantly reduce cumulative energy system costs
• ASEAN faces challenges from the gaps in infrastructure investment requirements

## Graphical abstract

**Figure:**
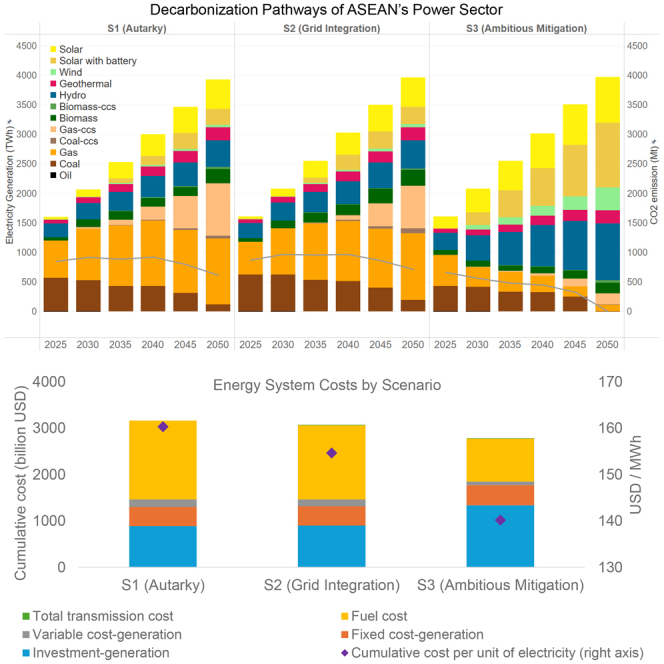

## Introduction

**Figure 1:**
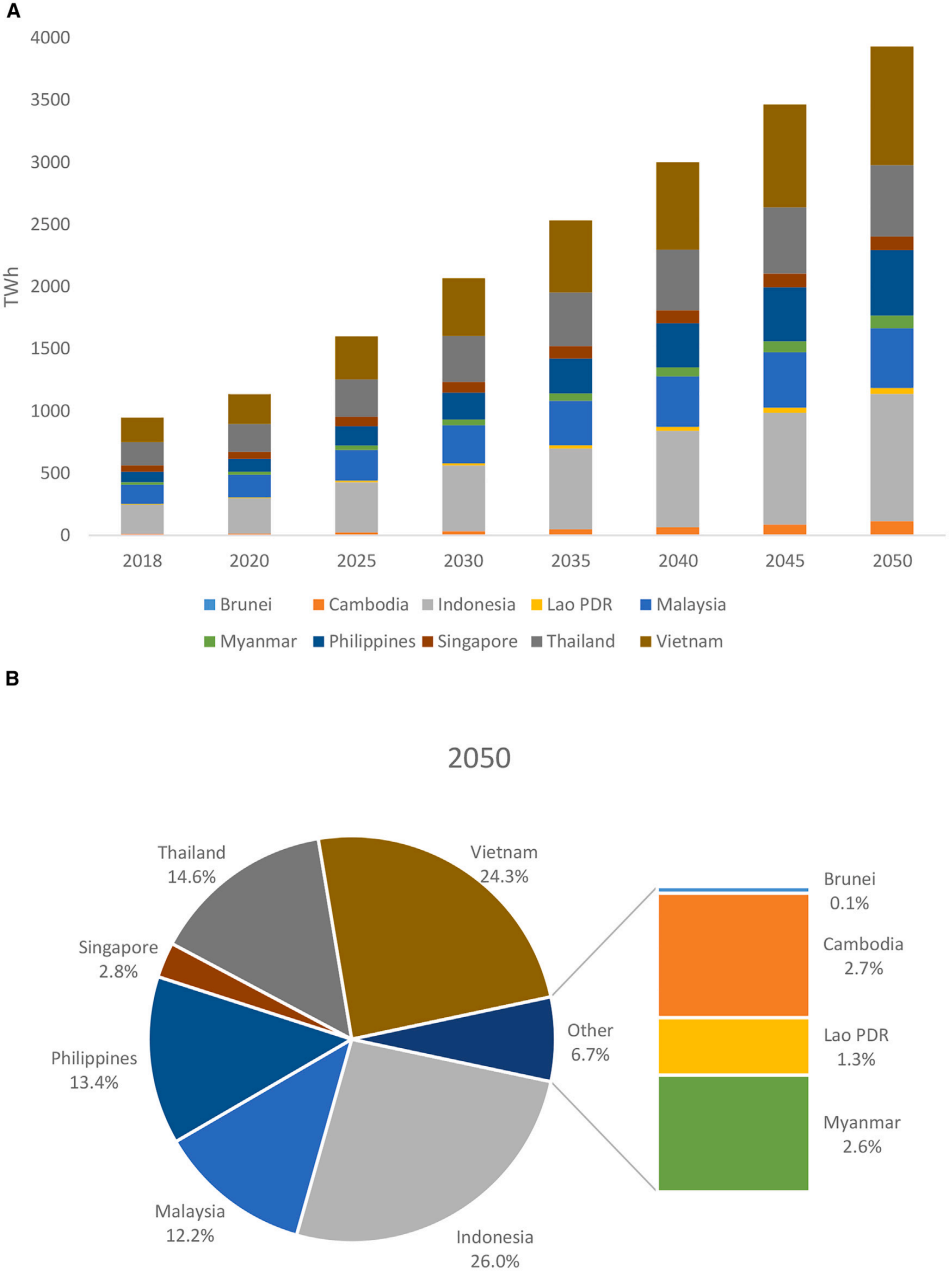
Projected electricity demand in ASEAN (A) Evolution of electricity demand by ASEAN countries. The projections are prepared by the authors (see supplemental information) and consistent with the IRENA projections.^8^ (B) The share of electricity demand by country in 2050.

It is well known that economic growth alone cannot lead to sustainable development given current trajectories of resource use and population growth.^1^ The energy transition, especially in the power sector, is critical. Motivated by increasing global momentum toward net zero, the ten countries of the Association of Southeast Asian Nations (ASEAN) are at a turning point. Due to burgeoning economical and demographical development, its electricity demand is projected to more than triple by 2050 relative to 2018 levels (Figure 1A). However, the region still relies heavily on electricity from fossil fuels, and has one of world’s fastest growth rates of emissions.^2^ Hydropower, mostly in the Lower Mekong region, is the only commonly used renewable resource (accounting for about 15.6% in the current generation mix), whereas other renewables, given ASEAN’s potentials, are under-utilized.^3^ To accelerate decarbonization, all ASEAN countries have announced their Nationally Determined Contribution (NDC) commitments under the Paris Agreement.^4^ There are several strategies available for the ASEAN power sector: cross-border power trade through the ASEAN Power Grid and directed technological change^5^^,^^6^ through the deployments of renewables and low-carbon technologies, particularly Carbon Capture and Storage (CCS).^7^ While these strategies are well known, how to incorporate them in an optimal and effective manner for ASEAN to better understand the evolution of power generation portfolios that can meet future electricity demand and 2030 NDCs and beyond requires rigorous analyses.

The fundamental purpose of such analyses is to design viable pathways for ASEAN’s power sector to meet future electricity demand and achieve various emissions targets, particularly a net-zero emissions target by 2050. These desired features, however, are not always incorporated into a unified modeling framework in the literature on ASEAN’s net-zero energy transition (see Table S12 for a detailed literature review). A strand of the literature applies approaches such as simulation,^3^ life cycle assessment,^9^ and policy assessment,^10^ which do not necessarily generate the optimal pathways. A key research gap in the literature is that the dynamics of power trade are not explicitly modeled.^3^^,^^11^ The availability of cross-border grids (and the expansion), however, can change historical trends and dispatch patterns in the power sector. Existing studies incorporating cross-border transmission in the energy system cost-optimization have conducted the analysis for a single modeling year instead of a long-run pathway to 2050,^12^^,^^13^ or based on a renewable target in generation (e.g., 100% renewables).^8^

The targeted modeling frameworks should cover all ten ASEAN countries, and capture the characteristics of various generation technologies, and the integration into transmission networks. Such strategic capacity planning models typically optimize generation portfolios over different locations under environmental constraints, e.g., grid emission intensity limits. These studies provide important information for climate policymaking, as they help better manage the trade-offs between fossil fuel-based and low-carbon generation technologies. However, these environmental objectives do not directly represent country-specific NDCs^14^ or incorporate a net-zero emissions target.^15^ More importantly, ASEAN is a region comprising heterogeneous countries that differ in terms of domestic circumstances, including electricity demand (Figure 1), ambition and ability to meet emissions targets in power sector planning,^16^ and renewable energy potentials such as solar and wind, geothermal^17^ and bioenergy. However, country-specific results of pathways for all ASEAN countries remain sketchy in literature.^18^

Here, we present a unified modeling framework that covers the energy transition strategies of all ten ASEAN countries to fill research gaps. Using the modeling tool URBS^19^ with country-level and annual temporal resolution, we minimize total power system cost for generation, transmission, and storage over the period 2018–2050. The scenario-based results are not a prediction of what will occur, but rather identify hypothetical pathways to achieve a low-cost future ASEAN power sector given a set of assumptions (e.g., geopolitical cooperation and sufficient investment) and technical parameters (supplemental information for details). A key challenge identified in the literature is ASEAN-specific data availability. We have collected diversified techno-economic data inputs that are specific to generation and transmission technologies, countries, fuel types, resource types and years. We also introduce country-specific emission constraints derived from their NDCs and the potential plans to further decarbonize the ASEAN power sector, instead of a common environmental constraint for all. We believe that these attributes result in a deeper understanding of decarbonization in the ASEAN power sector, as NDCs are set for each country on a yearly basis.

An important feature of this tool is that it can incorporate a directed transmission network, which allows us to model the power system’s spatial heterogeneity properly. This is important, because integration to regional blocs or global economy suggests the movements of economic activities,^20^^,^^21^ and also the environmental impacts.^22^ In particular, this tool allows for the cross-border reallocation of generation capacity and an understanding of the required supporting transmission capacity. The model is able to quantify the country-specific gap in investment required to achieve various emission targets and show that this gap is unevenly distributed across ASEAN countries. The spatial heterogeneity is a critical concern, although cross-border grids can be overall welfare-improving.

We run the model over the period 2018–2050, and identify different pathways that are able to meet ASEAN’s future electricity demand and achieve the emissions targets for 2030 and beyond, including a target of net-zero emissions. The results show that an emission-free power sector is achievable by 2050, and the implementation of the ASEAN Power Grid can provide a saving of up to 11.9% in terms of cumulative system cost. While it is clear that a transition to clean generation technologies must take place, the extent differs significantly across scenarios. With moderate emissions targets, fossil fuel-based generation technologies, including those with CCS, would still play a critical role, accounting for 53.7%–55.3% of ASEAN’s 2050 generation. However, achieving net-zero emissions requires an accelerated transition toward renewable energy, reaching 92% in ASEAN’s 2050 generation mix, of which solar power accounts for 47.2%. Meanwhile, ASEAN countries with lower GDP (Gross Domestic Product) per capita face a greater economic burden in developing the essential infrastructure to get on track to achieve their emissions targets.

## Results

### Capacity expansion plans

**Figure 2:**
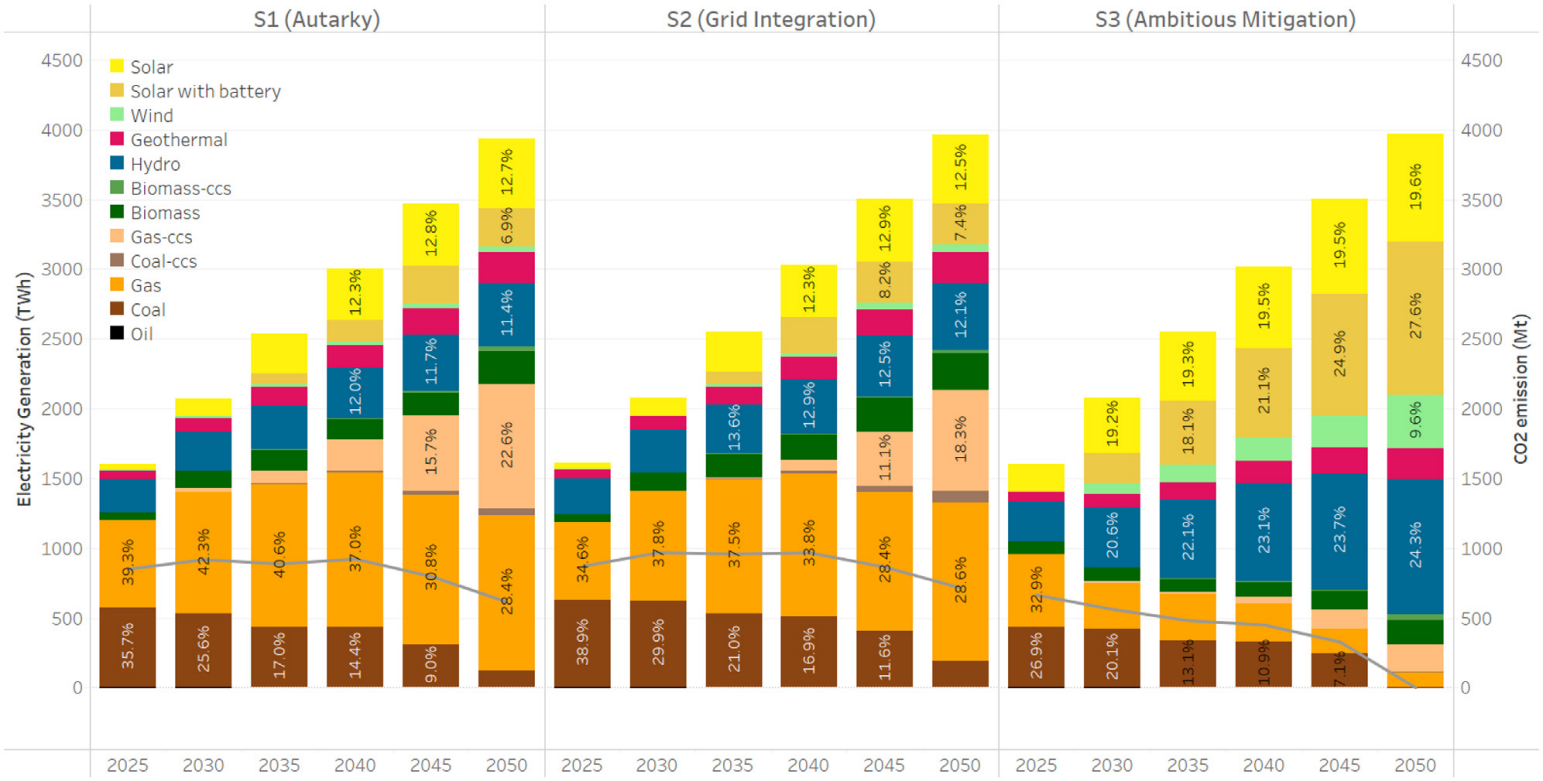
Generation mix of ASEAN We report the generation mix (aggregated across all ASEAN countries) for S1 (autarky), S2 (grid integration), and S3 (ambitious mitigation). The curves show the total power system’s CO_2_ emissions.

We perform a power system optimization at the national level for ASEAN, considering three scenarios (Table 1). Figure 2 shows the generation mix by technology through 2050 for ASEAN. The results provide useful information for policymakers to better manage the trade-offs between various energy transition strategies.

**Table 1:** Scenario description

Scenario	Description
S1 (autarky)	No cross-border transmission allowed. Moderate renewable resources. Moderate emissions targets (2030 NDCs and halve emissions by 2050).
S2 (grid integration)	Planned transmission network and capacity expansion allowed. Moderate renewable resources. Moderate emissions targets (2030 NDCs and halve emissions by 2050).
S3 (ambitious mitigation)	Planned transmission network and capacity expansion allowed. Resource exploration with higher hydro, solar and wind potentials. Low emissions targets (2030 NDCs and zero emissions by 2050).

All ASEAN countries are included in the planned transmission network, in which net electricity imports can account for up to 60% of domestic electricity demand, for example, for Singapore.^23^ The limits of electricity imports and exports vary across countries (see the scenario development section in supplemental information). Moderate renewable resources mostly refer to those announced in the policy-oriented documents, for example, in the country’s Biennial Update Reports to the UNFCCC (United Nations Framework Convention on Climate Change), and are typically smaller than those from technical assessments.^24^ The moderate emissions targets in this study are consistent with the Announced Pledges scenario for Southeast Asia in the IEA (International Energy Agency)'s World Energy Outlook series.^18^^,^^25^

The pathways toward moderate emission targets rely heavily on fossil fuels (oil, gas, and coal) to satisfy electricity demand. This finding coincides with the reports by IRENA (International Renewable Energy Agency)^8^ and IEA (International Energy Agency),^18^^,^^23^ where current stated policies are assumed. In S1 and S2, the use of unabated fossil fuels is projected to peak in 2040, with coal and gas contributing up to 51% of total generation. The share of unabated fossil fuels declines afterward, with unabated coal falling to less than 5% and gas falling to around 29% by 2050. An important pattern is the drastic transition from unabated fossil power to CCS technologies between 2030 and 2050, with CCS accounting for around 24% of total generation in 2050. Gas-CCS plays a key role in meeting ASEAN’s moderate decarbonization targets because of its lower CO_2_ emissions and lower cost than coal-CCS. Total share of fossil fuel-fired CCS in this study is consistent with that estimated in IRENA’s findings, in which CCS accounts for about 25% of total generation in 2050.^8^

On the other hand, the moderate decarbonization goals in S1 and S2 require over 44% of renewables in total generation. While hydropower and geothermal capacities expand steadily in each modeled year, reaching approximately 12% and 6% of total generation, respectively, solar power gradually becomes the dominant renewable energy source in ASEAN, even accounting for higher battery costs.

In the absence of an ambitious decarbonization target, grid integration does not significantly alter the generation mix in S2. However, it does encourage greater utilization of renewable resources, such as biomass, hydropower, solar and wind. Gas-CCS also enters generation mix in S2 at a later stage in order to reduce costs.

However, to achieve the net-zero emissions target, S3 depicts a significantly different decarbonization pathway. An accelerated transition from fossil fuels to renewable energy sources will drive the renewable energy penetration level to 92% in 2050, with minimal expansion of thermal power plants. The 2050 goal profoundly impacts the ASEAN power system even in the early stages, with renewable penetration exceeding 60% as of 2030, indicating a rapid expansion of renewable power generation will be needed in the near term. Solar power generation amounts to 615 TWh in 2030 and grows steadily thereafter to a total of 1,875 TWh in 2050. Wind power will emerge in 2030 and play a much more significant role than in S1 and S2.

While the generation mix is similar to other net-zero power sector studies for ASEAN that suggest a 100% renewable energy generation mix,^8^^,^^11^^,^^14^ it should be noted that in the S3 scenario fossil fuels still constitute about 8% of the total generation in 2050. These are primarily utilized in gas-CCS power plants (5%) and gas power plants without CCS (3%). The installation of negative emission technologies, such as biomass-CCS, offsets the utilization of positive emission thermal power technologies.

### Heterogeneity across countries

**Figure 3:**
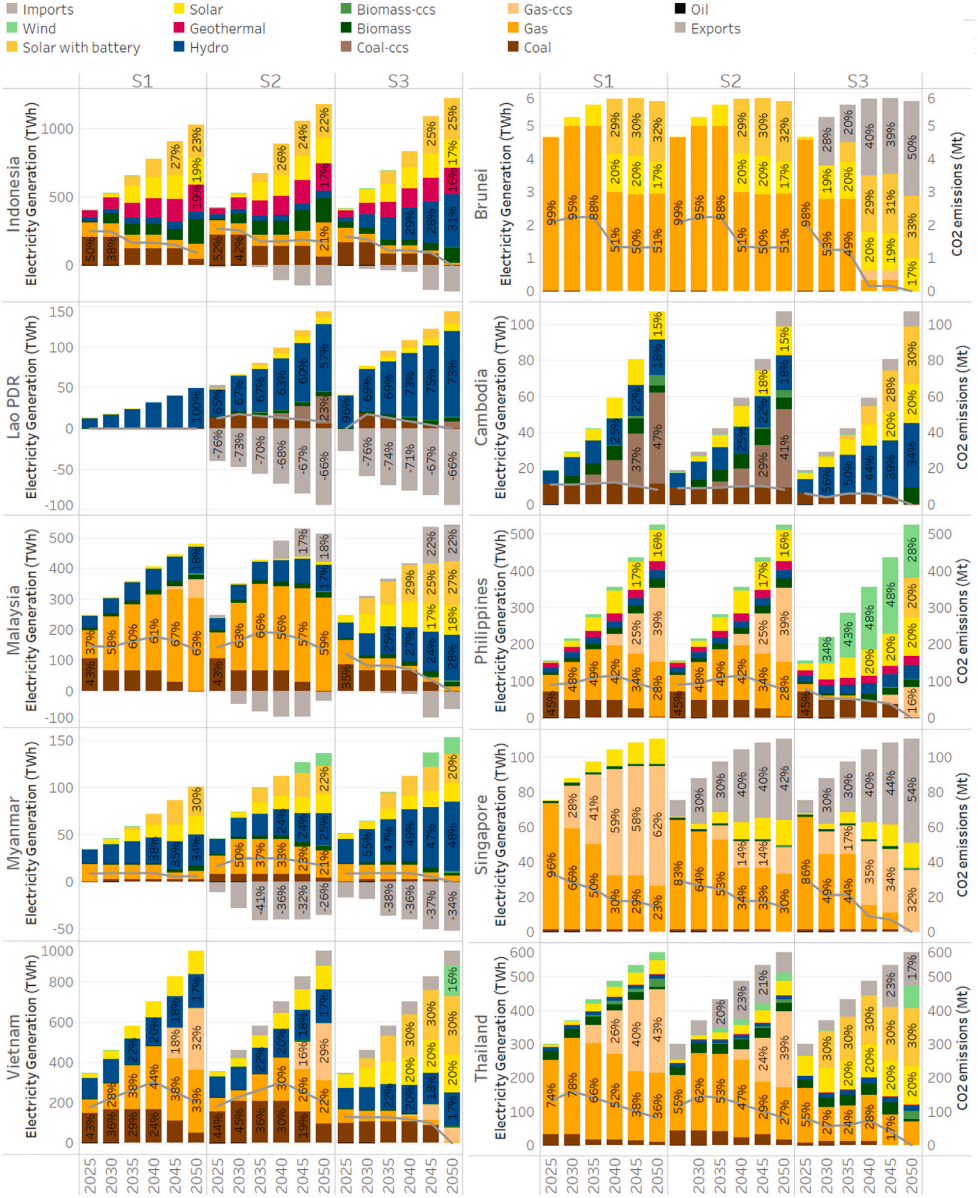
Generation mix by country Evolution of power generation for S1 (autarky), S2 (grid integration), and S3 (ambitious mitigation) from 2025 to 2050. The stacked bar chart shows the pathway of generation mix for each country (left axis), and the gray curve shows the CO_2_ emission in kilo tonnes (right axis). The percentage marks the share of electricity generation in total electricity generation.

The decarbonization pathways vary depending on each country’s natural energy endowment, during the transition away from unabated fossil fuels (Figure 3). Meeting the increasing electricity demand in a sustainable manner can be a significant challenge to ASEAN countries in the less favorable scenarios in which renewable resources are limited. Therefore, this research identifies the potential for CCS technology to support the region’s transition. Under a moderate emissions reduction target, coal-based CCS could be adopted as early as 2035 in Cambodia and 2040 in Lao PDR. Gas-CCS produces lower emissions and is thus favored by Malaysia, the Philippines, Singapore, Thailand, and Vietnam. However, if ASEAN aims to achieve net-zero emissions by 2050, the adoption of CCS would be less favorable, with only a few countries adopting it at a later stage and on a much smaller scale. The reasons are 2-fold: firstly, exploring the technological potential of renewable resources could provide these countries with more affordable and available power resources; secondly, the positive emissions that need to be offset by biomass-CCS make them less economically attractive.

Hydropower is one of the most common clean energy sources in ASEAN, and it is well exploited wherever available, specifically in the Great Mekong subregion, Malaysia, Indonesia, and the Philippines. On the other hand, solar power is widespread in ASEAN. Endowed with great solar potential, solar plays a central role in reaching carbon-free goals in most countries, especially those without sufficient hydropower potential. The results suggest that by 2050 most ASEAN member states will have more than 40% of the generation mix composing of solar power by 2050. Wind power is introduced in the Philippines, Myanmar, Thailand, and Vietnam due to its limited availability.

Enabling transmission will re-distribute natural resources and increase the use of solar power and hydroelectricity, especially in Myanmar and Lao PDR. Lao PDR is expected to be the largest clean energy exporter in both scenarios (S2 and S3) in terms of generation mix shares. The results also suggest that Indonesia may potentially become a major exporter in terms of export volume. By importing clean energy, Thailand, Vietnam, Brunei, and Singapore avoid adding excessive fossil fuel power capacity and also delay the investment in CCS technologies. In addition, clean energy imports play a key role in these countries to their net-zero goals.

The timing of peak emissions differs in each scenario and country. Under the moderate decarbonization target, emissions peak in around 2040 for ASEAN, while many countries are projected to reach the peak later in 2035 and 2040 as fossil fuel usage diminishes. However, if ASEAN aims for a carbon-free power system, immediate actions are necessary and peak emissions would be significantly lower than those allowed by the moderate emission targets.

### Enabling cross-border transmission grids

**Figure 4:**
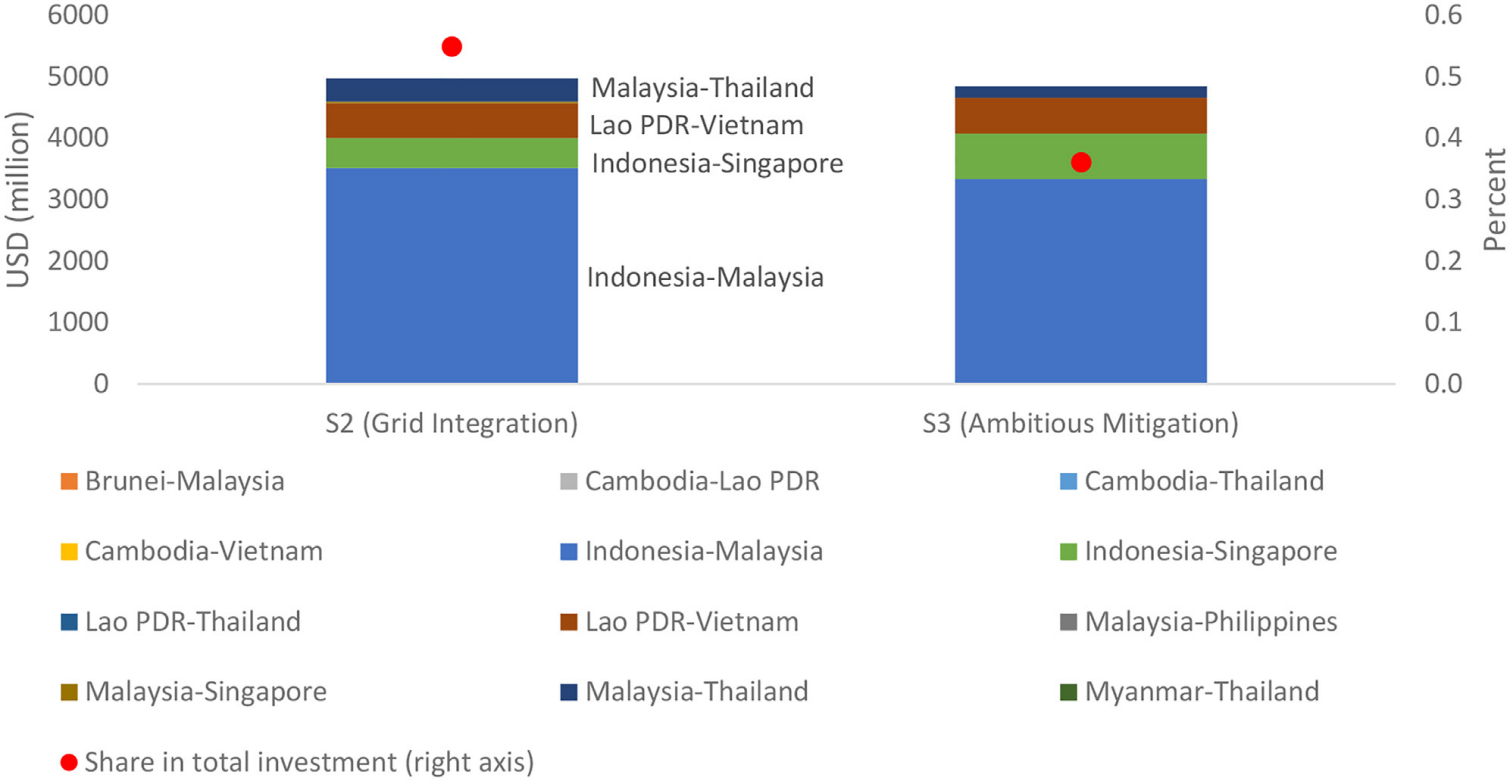
Infrastructure investment in transmission lines We report the cumulative investment for each transmission line over all model years (left axis) and share in total infrastructure investment (i.e., generation and transmission) on the right axis.

This study identifies the most important transmission lines from an investment point of view (Figure 4). While total infrastructure investment in transmission is 5.0 billion USD in S2 and 4.8 billion USD in S3, transmission investment only accounts for a marginal share of total infrastructure investment (0.5% and 0.4%, respectively). For the entire power system to operate effectively, a small number of transmission lines requires the most investment. In other words, underinvestment is an issue for these transmission lines. Identifying these transmission lines can provide useful information for infrastructure planning.

In S2, three transmission lines, i.e., Malaysia–Thailand (0.4 billion USD), Lao PDR–Vietnam (0.6 billion USD), Indonesia–Singapore (0.5 billion USD), and Indonesia–Malaysia (3.5 billion USD), are critical, accounting for 98% of total investment in transmission. In S3, those four key transmission lines remain important in terms of the investment requirement.

### Economic impacts

**Figure 5:**
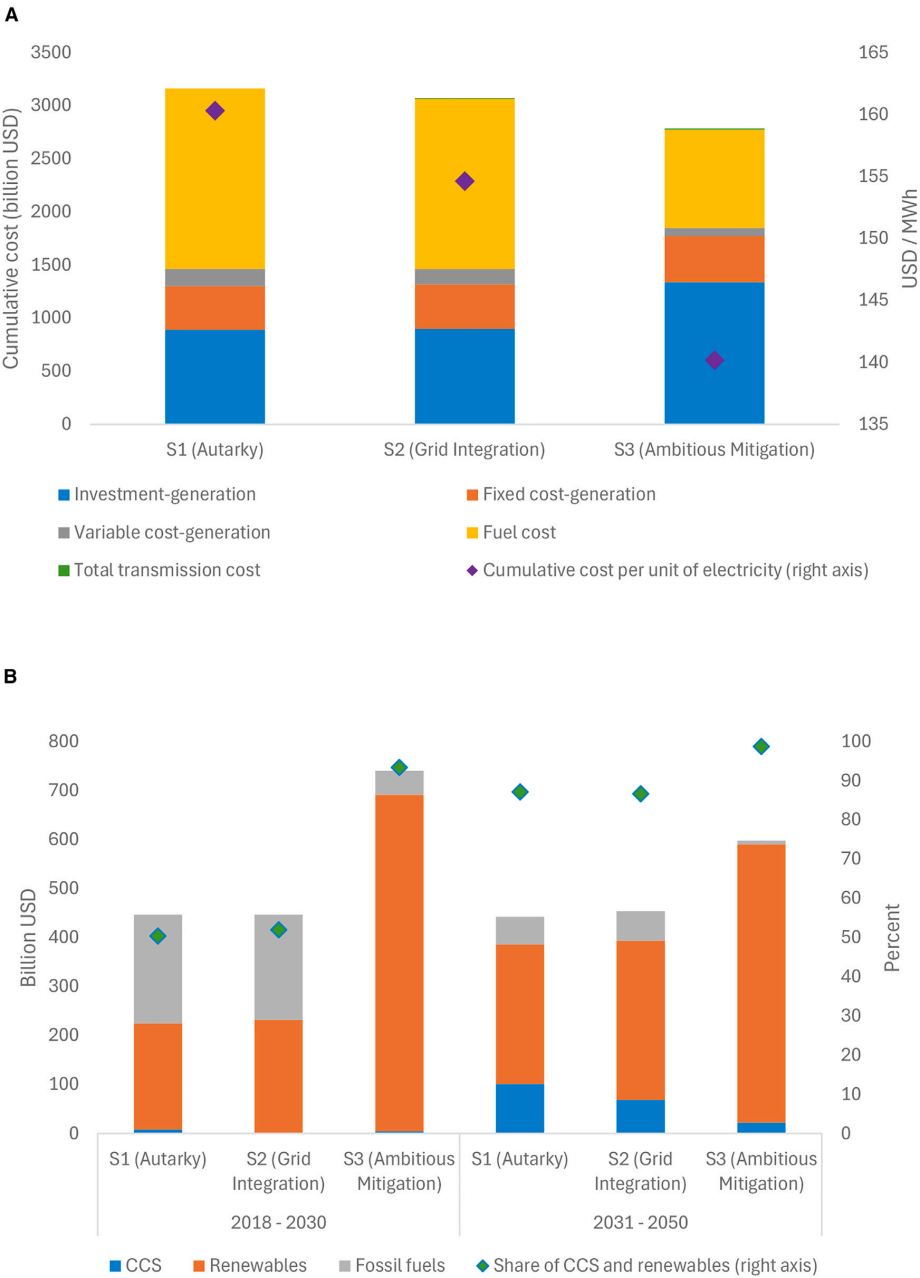
Analysis of economic impacts Results are aggregated over all ASEAN countries. (A) Cumulative system cost by type (left axis) and cumulative unit cost of electricity (right axis) are presented. Total transmission cost includes investment and fixed cost in transmission. (B) Cumulative infrastructure investment in generation by technology by period (left axis) and share of CCS and renewables in total generation investment (right axis) are presented.

Our findings further show the economic impacts on energy system costs and infrastructure investment in generation. In the scenarios where cross-border transmission is allowed, total transmission cost still accounts for a marginal share in cumulative system cost, i.e., 0.3% in S2 and 0.32% in S3. Although transmission costs take up a marginal share, cross-border transmission leads to a much larger share of economic benefit (Figure 5A). This highlights the economic benefits of cross-border power trading and cooperation for the development across the ASEAN region and the achievement of emissions targets as a whole. As compared to a “go it alone” scenario (S1), cumulative system cost drops by 2.8% in S2 and 11.9% in S3. The unit cost of electricity declines from 160 USD/MWh in S1 to 155 USD/MWh in S2 (−3.6%), and to 140 USD/MWh in S3 (−12.6%). S3 requires the most investment in generation, while fuel cost in generation greatly diminishes as an accelerated transition toward renewable energy takes place. Our findings are complementary to the study of net-zero energy transition in China, which highlights the reduction in abatement costs arising from the expansion of solar and wind power, domestic transmission, and storage.^24^

Figure 5B further investigates the structure of generation investment. From 2018 to 2030, traditional fossil fuel-based generation still plays an important role in investment portfolio in S1 and S2 (CCS and renewables accounting for 50% and 52%, respectively). However, investment in CCS is not needed in S2 at an earlier stage. A fully connected cross-border transmission network under S2 can substitute CCS. For S3, substantial investments in renewables are required, and they need to begin earlier. In the post-2030 period, CCS and renewables would account for nearly 100% of generation investment. This is in line with the IEA outlook study for Southeast Asia.^23^

This study quantifies the generation investment by country (Table 2). For the entire ASEAN, the cumulative infrastructure investment in generation accounts for 29.6%–44.6% of ASEAN’s GDP in 2018 across scenarios. This points toward the need for significant financing to support the green transition for the power grid. In particular, the findings also suggest that there is heterogeneity in investment gaps across countries, with financing more urgently required in countries in the Greater Mekong Subregion and the Indonesian archipelago where there are more abundant sources of renewable energy but a significant lack of infrastructure and capital.

**Table 2:** Cumulative infrastructure investment in generation by country

Country	S1 (autarky)	S2 (grid integration)	S3 (ambitious mitigation)
Brunei	1.8	1.8	1.6
Cambodia	22.0	19.8	25.8
Indonesia	281.5	316.4	330.5
Lao PDR	3.6	27.4	33.8
Malaysia	95.2	99.5	173.9
Myanmar	22.1	36.6	44.9
Philippines	98.9	99.0	175.4
Singapore	28.9	16.5	18.8
Thailand	157.2	118.8	212.0
Vietnam	177.4	164.3	321.1
ASEAN	888.7	900.1	1337.8
% in 2018 GDP	29.6	30.0	44.6

Investment is expressed in billion USD. Total investment is compared to ASEAN’s 2018 GDP.^26^

Under moderate emissions targets, cross-border transmission does result in investment gaps in 5 countries, i.e., Indonesia, Lao PDR, Malaysia, Myanmar, and Philippines, all of which are emerging and less developed countries. A typical example is Lao PDR, in which the investment required sharply increases from 3.6 billion USD (S1) to 27.4 billion USD (S2), and to 33.8 billion USD (S3). The investment gaps also highlight the geographical differences in supply and demand centers across ASEAN that require multilateral cooperation to overcome.

Comparing S2 and S3, the investment gaps become larger for most countries in order to achieve net-zero emissions, except for Brunei. The task of enhancing the power grid interconnections and capacity is further hampered by differences in levels of development, on top of differing national energy policies and regulations relating to the power grid. Major emerging ASEAN countries, such as Indonesia, Malaysia, Philippines, Thailand, and Vietnam, face a greater investment requirement in developing their domestic infrastructure and will need significant foreign investment to realize their projected potential.

## Discussion

Our study identifies different pathways that are able to meet future electricity demand and achieve various emissions targets, including a net-zero emissions target by 2050. The results show that an emission-free power sector is technically achievable under the assumptions of this model. Several key findings stand out.

(1) Fossil fuels (including those with CCS) will still play a critical role in the region’s generation mix through 2050 under moderate emissions targets. The results highlight the key role of CCS in meeting emissions targets in less favorable situations with moderate renewable sources. This can be an opportunity for ASEAN^25^^,^^27^ to move more rapidly as a region in terms of technology and governance. National and regional legal and governance frameworks for domestic and cross-border CO_2_ transportation and storage are needed.^7^
(2) Achieving an emission-free power sector in 2050 requires an accelerated transition toward renewable energy, leading to a markedly different pathway from the other two scenarios. In such a scenario, fossil fuel-based power generation declines, accounting for only 8% of the 2050 generation mix (including those with CCS). To facilitate investments in renewables in ASEAN, it is critical to overcoming the barriers in renewable energy legislation, energy governance, and business environment.^28^
(3) Cooperation through the ASEAN Power Grid brings economic benefits to the region as a whole, and thus improves the affordability for energy transition. This provides useful quantitative information supporting the key policy message addressed by the IEA.^18^ Compared to the “go it alone” scenario (S1) in which regional interconnections are not allowed, this brings considerable total benefits by reducing cumulative system cost and cumulative unit cost, for example, by 11.9% and 12.6% respectively, and achieving net-zero emissions.
(4) The realization of the ASEAN Power Grid faces challenges from the gaps in infrastructure and its corresponding investment requirements. Our results are able to quantify such country-specific gaps and highlight the heterogeneity of financing needs across ASEAN for the realization of the ASEAN Power Grid. This is important for developing and updating the regional energy interconnectivity master plan.^26^

Realizing the massive infrastructure construction, especially in the Greater Mekong Subregion, calls for urgent action to ensure adequate financial resources and political support. While the economically optimal pathway can be pictured through our model, the heterogeneity of gaps in infrastructure underscores the unique and mammoth challenge that ASEAN faces, as a group of sovereign countries with significant differences in development, national policies and socio-political systems, in realizing an integrated regional power grid. This highlights the need for a just transition and benefit sharing. Solutions required go beyond financing, foreign investments and public-private partnerships. There is a need to explore suitable multilateral mechanisms for greater regional cooperation (e.g., the ASEAN Economic Community and ASEAN Plus Three), information sharing, and improvements in technical coordination.^29^

### Limitations of the study

Our capacity expansion modeling framework focused primarily on the technical feasibility of achieving predefined emission targets at minimum cost for ASEAN as a whole. While scenarios 2 and 3 assumed a degree of cooperation and country-specific import and export limits as a proxy for energy security goals, this study did not address other factors that complicate the realization of such objectives. For example, institutional barriers, such as subsidies for fossil fuels,^30^ complicated regulatory environment,^31^ inefficient market mechanisms and lack of investment^32^ are challenges to deploying renewable energy and greater cross-border power trading. In addition, for emerging technologies such as CCS, the techno-economic data are based on current assessments. This study did not consider the uncertainties in the cost assumptions and long-run trajectories. For example, the cost of CO_2_ transportation and storage can significantly vary across projects in ASEAN.^33^ Further research is needed on these topics.

## Resource availability

### Lead contact

Further information and requests for code may be directed to the lead contact, Bin Su (subin@nus.edu.sg).

### Materials availability

This study did not generate new unique reagents.

### Data and code availability

• The data used in this study are obtained from various sources (see data in method details, and Tables S1–S11 and Figures S1–S4 in supplemental information).
• The URBS modeling tool^19^ (with Python 3.7.1) was modified and used to perform the modeling in this paper. See supplemental information document for details of the model modification.
• Any additional information regarding the data and analysis reported in this paper is available from the lead author upon reasonable request.

## Acknowledgments

This work was supported by ExxonMobil through the 10.13039/501100022754Singapore Energy Centre (EM11161.TO12). We would like to thank Dr. Adam Usadi, Dr. Michael Harper, Prof. Ang Beng Wah, Prof. Lee Poh Seng, Dr. Christopher Len, and Dr. Goh Tian for their helpful support.

## Author contributions

B.S. and D.J.P. designed the research, S.Z. and L.Y. processed the data and built the optimization model, and all authors contributed to the formal analysis and paper writing.

## Declaration of interests

The authors declare no competing interests.

## STAR★Methods

### Key resources table

**Table:** 

REAGENT or RESOURCE	SOURCE	IDENTIFIER
**Deposited data**		

Original dataset	This paper	https://doi.org/10.2139/ssrn.4605624

**Software and algorithms**		

URBS	Dorfner^19^	https://urbs.readthedocs.io/en/latest/
Python 3.7.1	Python Software Foundation	https://www.python.org/downloads/release/python-371/
Tableau	Salesforce	https://www.tableau.com

### Experimental model and study participant details

There are no experimental model or study participants to include in this paper.

### Method details

#### Data

The data used in this study are collected from various sources. Each ASEAN country is viewed as a power system with country-specific electricity demand and emission targets required by its NDC. We construct an ASEAN-specific dataset of techno-economic information for all generation technologies (Table S1), starting with microdata at the power plant level for ASEAN.^34^ This includes investment cost, fixed O&M cost, variable cost, efficiency, lifetime and grid emission factor. We also obtain the installed capacity, remaining lifetime, and capacity factor by technology and country. Coal with CCS and natural gas with CCS are assumed to have a 90% capture, while biomass with CCS is introduced as a carbon-negative technology with a negative grid emission factor.^35^ CCS technologies are expected to be viable from 2030 and onwards and their techno-economic characteristics are specific to ASEAN.^11^^,^^36^ The cost of CO_2_ transportation and storage is assumed to be USD 20 / tonne CO_2_.^7^ We assume that new coal-fired power plants without CCS will be prohibited after 2030. At COP26 in 2021, countries such as Indonesia, Malaysia, Philippines, Singapore and Vietnam made new commitments of phasing out coal power.^37^ No New Coal strategy is also included in Cambodia’s long-term climate strategy.^38^

Increasing R&D and deployment lead to radical innovations,^39^ which, in turn, would lower the investment cost of emerging generation technologies.^40^^,^^41^ A typical example is solar PV technology.^42^ We consider the investment cost reduction for coal with CCS, natural gas with CCS, solar PV, solar PV with battery and wind, following technology-specific growth rates.^43^ In ASEAN, the efficiencies of coal and natural gas are relatively low,^44^ and we assume both can achieve the levels of Asian economies like China and India.^45^ The solar PV capacity factor improvement by country is derived using the PVGIS platform developed by the Joint Research Centre.^46^ We further assume that the total share of solar PV (with and without battery) in domestic consumption is limited to 50%,^8^ and the maximum share of solar PV without battery is 20%.^47^

Thailand’s residual fuel oil price is used as the representative ASEAN fuel oil price in 2018,^48^ while the price projection is derived using the price growth rates from the World Bank^49^ and the US Annual Energy Outlook.^50^ Australian coal price and Japan's natural gas price are used as the benchmark prices in this study. Historical coal prices are collected from the World Bank^49^ and the projections are obtained from the McCloskey Coal Price Outlook.^51^ Natural gas price projections up to 2024 are taken from the World Bank,^49^ and the projections for 2030 and 2050 are based on the assumptions from the IEA Global Energy and Climate Model.^52^ The natural price projections between 2024 and 2030, and between 2030 and 2050 are then interpolated. For biomass, we first estimate the price in 2018. We consider ASEAN’s average price of biomass materials, i.e., USD 20 / tonne,^53^ and five types of biomass materials, i.e., forest residues, wood waste, agricultural residues, energy crops and landfill gas.^54^ Biomass price trajectories to 2050 are derived using fuel-specific price growth rates.^55^

We distinguish between the moderate level of resource potentials and those higher potentials based on technical assessments for hydro, solar and wind (Table S6). Due to the impacts of dams on hydrological regimes^56^ and fish biodiversity,^57^ hydro potential may not be fully utilized and the moderate hydro potential is interpolated based on historical deployment data from IRENA.^58^ For each ASEAN country, we collect solar and wind technical potentials as well as those moderate (also smaller) potentials from policy-oriented documents.

All countries are connected by transmission lines (HV onshore and HV offshore). The network structure and initial transmission capacity are based on prior studies, and the collected data are in line with the energy outlook of the ASEAN Centre for Energy.^3^ With the unit cost of transmission,^59^ we obtain the techno-economic data for transmission by incorporating capacity, length and type of transmission (Table S8). The length of the transmission is collected from literature where available or calculated as the great circle distance between two connecting locations.

#### Capacity expansion model

The optimization model is formulated as a linear programming model with perfect foresight that gives a cost-optimal pathway for the power sector (see supplemental information for details). Specifically, the objective minimizes the net present value of the total ASEAN system cost to expand and operate the power system that can meet the electricity demand, including the annualized investment costs for both electricity generation and transmission infrastructure, fuel costs, and fixed and variable operations and maintenance costs, given the techno-economic information. The other model inputs are projected electricity demand, renewable targets, emission limits, and exogenous attributes associated with any prospective technology in each member state, such as initial capacity and maximum potential, emission intensity and emission targets, and so on.

The decision variables of the model involve expansion decisions and system operation decisions. For expansion, two types of assets, namely electricity generation infrastructure and transmission lines, will be expanded over the time horizon. Time intervals of 5 years are considered in the model, and we assume the expansion decisions are made once per time interval and will be ready at the beginning of each time interval, regardless of the lead time for construction. We calculate the total available capacity of an asset as the capacity that is fully operational during the whole-time interval, and an asset retires after reaching its lifetime. The total available capacity is upper-bounded as per the natural resource potential. Specifically, solar PV and solar PV with battery share the same resource potential in each country; and when solar PV with batteries becomes widely approachable from 2030 on, we prevent the capacity expansion for solar PV without batteries. The total available renewable generation capacity must meet a renewable target as well.

The system operation decisions include the electricity generation by each technology, the power transmission between neighboring countries, the total consumption of fuels and total carbon emissions. The output of electricity and net import must satisfy the projected electricity demand for each country. The throughput of each technology is subject to the total available capacity and the maximum capacity factor. In addition, for biomass and biomass-CCS, the throughput is also restricted by the projected maximum number of available biofuels for each nation annually. The fuel consumption and CO_2_ emission are subject to the heat rate and emission intensity, respectively, and a negative emission intensity offsets the total annual emission.

The model explicitly determines the capacity expansion and power flow for the transmission lines of the ASEAN Power Grid, and each line connects two neighboring countries. Electricity can be transmitted from one member state to another via the grids across multiple countries, and the electricity loss multiplies to reflect the higher transmission loss from long-distance transmission. The flow on each transmission line is not only limited by the transmission line capacity, but also by a restriction on power trade limit. A maximum import limit restricts the proportion of net imports to a country's domestic demands so that it prevents a country from being entirely reliant on electricity imports.

In this study, we set an annual CO_2_ emission allowance for each country separately from 2030, according to the NDC goals. Unlike many other studies that set a constraint for emission targets for ASEAN as a whole, a country-wide emission allowance reflects the heterogeneity of ASEAN member states' decolonization pledges.

### Quantification and statistical analysis

There are no quantification or statistical analyses to include in this paper.
