# Supplementary material for: Accelerating ASEAN’s energy transition in the power sector through cross-border transmission and a net-zero 2050 view
Source: iScience. 2024 Dec 6;28(1):111547. doi: 10.1016/j.isci.2024.111547 (PMC11719848; doi:10.1016/j.isci.2024.111547)
Supplement: Document S1. Figures S1–S4, Tables S1–S12, and Data S1 [file mmc1.pdf]

iScience, Volume *28*

Supplemental information

Accelerating ASEAN's energy transition in the power sector through cross-border transmission and a net-zero 2050 view

Sheng Zhong, Lingyi Yang, Dimitri J. Papageorgiou, Bin Su, Tsan Sheng Ng, and Saifudin Abubakar

- **Data and Methods S1: Supplemental Information on Data Collection and Mathematical Model [Related to STAR Methods]**

Power Generation Technologies

In this study we consider 12 generation technologies, five of which are fossil fuel-based. Three types of Carbon Capture and Storage (CCS) technologies are included, i.e., coal with CCS, natural gas with CCS and biomass with CCS. The characteristics of all generation technologies in 2018 are summarized in Table S1.

Based on a micro-dataset at the power plant level (Enerdata, 2021), we estimate the ASEAN-wide average investment cost for the following generation technologies: oil, coal, natural gas, hydro, geothermal and biomass. The average costs are expressed in 2018 US dollars. The estimates in this study mostly fall within the cost ranges reported in Handayani, Anugrah, Goembira, Overland, Suryadi, and Swandaru (2022).

The country-specific investment costs for solar PV and wind are obtained from ACE (2019b), which are also used as the default data inputs in NREL's RE Explorer tool (NREL, 2022). The country-specific investment costs are then expressed in terms of average investment costs over all ASEAN countries. According to NREL (2021), the investment cost of solar PV with battery is 44.9 percent higher than that of solar PV in base year. We assume that total share of solar PV (with and without battery) in domestic demand in each year should not exceed 50% (IRENA, 2022) and the maximum share of solar PV without battery is 20% (Albertus, Manser, & Litzelman, 2020), while the share of total renewables can still be higher than 50%.

For the three types of CCS technologies, the investment costs are ASEAN-specific, taken from Handayani et al. (2022); MEMR, Danish Energy Agency, and Ea Energy Analysis (2021). We assume that CCS technologies can only be applied from 2030 and onwards. New coal-fired power plants without CCS will be prohibited after 2030. At COP26 in 2021, countries such as Indonesia, Malaysia, Philippines, Singapore and Vietnam made new commitments of phasing out coal, including the "No New Coal" commitment by Malaysia, Philippines and Vietnam (UNFCCC, 2021). A similar "No New Coal" strategy is included in Cambodia's long-term climate strategy (Kingdom of Cambodia, 2020).

Fixed O&M cost in this study is represented by a percentage of investment cost. The fixed O&M cost of solar PV with battery is based on NREL (2021), while for all other renewables, the costs are obtained from Tsiropoulos, Tarvydas, and Zucker (2018). The fixed O&M costs of CCS technologies are taken from MEMR, Danish Energy Agency, and Ea Energy Analysis (2021), while for all others the costs are based on Huber, Roger, and Hamacher (2015).

For the generation technologies without CCS, the variable costs are taken from Handayani et al. (2022); Stich and Massier (2015). For coal with CCS and natural gas with CCS, we calculate the variable cost using the change in cost in comparison to the power plants without CCS (NREL, 2021)[1], and then add the cost for CO_2 transportation and storage. We use USD 20 / tonne CO_2 as the CO_2 transport and storage cost, based on IEA (2020a). Following the

[1] According to NREL's Annual Technology Baseline, the variable cost of coal with CCS would be 81.4 percent higher in comparison to coal without CCS. With this information, we obtain the ASEAN-specific variable cost for coal with CCS. Based on the same data source, for natural gas, the variable cost would increase by 229 percent if CCS is implemented.

same approach, the variable cost of biomass with CCS is estimated using the data in Sanchez, Nelson, Johnston, Mileva, and Kammen (2015).[2]

The thermal efficiency for oil, coal and natural gas is taken from the study on ASEAN's power plants (Goh, Ang, Su, & Wang, 2018), and the thermal efficiency for biomass is obtained from Huber, Roger, and Hamacher (2015). These data inputs on thermal efficiency are also consistent with the ASEAN average estimates reported in Paltsev, Mehling, Winchester, Morris, and Ledvina (2018). Due to the application of CCS technology, there is a loss in power plants' thermal efficiency. In this study, we estimate such a loss in thermal efficiency by comparing the heat rate of power plants with CCS to that of power plants without CCS. For coal with CCS and natural gas with CCS, the thermal efficiency drops to 26.22 percent and 37.89 percent, respectively (NREL, 2021).[3] For biomass with CCS, the thermal efficiency decreases to 26.81 percent (Sanchez et al., 2015).[4]

The lifetime of those non-CCS generation technologies is taken from various sources (ACE, 2019b; Huber, Roger, & Hamacher, 2015; Stich & Massier, 2015). The lifetime of CCS technologies is identical to those without CCS. This is consistent with NREL (2021), in which the depreciation period is identical for technologies with and without CCS if the same fuel is used.

The ASEAN-wide grid emission factors for oil, coal and natural gas are calculated using the data on power generation and CO_2 emissions from the IEA (IEA, 2021a, 2021c). For coal with CCS and natural gas with CCS, we assume a 90 percent capture, and thus the grid emission factors are 10 percent of those without CCS. This is in line with the settings in NREL's Annual Technology Baseline (NREL, 2021). According to Sanchez and Kammen (2016); Sanchez et al. (2015), grid emission factor for biomass is 0, and for biomass with CCS is a negative value.

Table S1. Characteristics of power generation technologies in 2018 [Related to STAR Methods]

Technology	Investment (USD / kW)	Fixed O&M cost (% of investment cost)	Variable cost (USD / MWh)	Efficiency (%)	Lifetime (years)	Grid emission factor (kg CO_2 / kWh)
Oil	1,310	3.52	2.5	34.11	30	1.28
Coal	1,551	4.00	4.0	33.52	40	0.99
Natural gas	1,042	3.52	2.3	42.63	40	0.45
Hydro	1,597	0.50	0	N.A.	50	0
Geothermal	3,001	2.00	0	N.A.	30	0
Solar PV	1,487	2.30	0	N.A.	20	0
Solar PV with battery	2,155	1.81	0	N.A.	20	0
Wind	2,168	3.00	0	N.A.	20	0
Biomass	2,322	2.00	6.0	35.00	20	0
Coal-CCS	3,470	2.84	25.08	26.22	40	0.10

[2] For biomass, implementation of CCS would lead to an increase of about 44.3 percent in variable cost.
[3] The heat rate of coal-fired power plants would increase from 8.473 to 10.834 MMBtu/MWh, if CCS is introduced. This means the efficiency of coal with CCS will drop by 21.79 percent. For natural gas plants, the heat rate would increase from 6.363 MMBtu/MWh (without CCS) to 7.159 MMBtu/MWh (with CCS), suggesting a decline of 11.12 percent in efficiency. We use this percentage change in this study, instead of using the heat rate values.
[4] The biomass power plant heat rate (12.5 MMBtu/MWh) would increase to 16.32 MMBtu/MWh, meaning a decline of 23.41 percent in efficiency. This percentage change is used to calculate the efficiency of biomass with CCS in ASEAN.

Natural gas-CCS	1,840	1.77	15.64	37.89	30	0.04
Biomass-CCS	5,453	1.17	34.86	26.81	20	-1.31

Note: N.A.: Not applicable.

In this study, we consider four types of emerging technologies that are perceived to have significant declines in investment costs over time, namely, coal with CCS, natural gas with CCS, solar PV, solar PV with battery and wind. For biomass with CCS, the investment cost is projected to be unchanged over time in the reference settings, according to Tsiropoulos, Tarvydas, and Zucker (2018). Figure S1 below plots the investment cost trajectories of the four emerging technologies. The cost projections are obtained using the moderate scenarios provided by NREL (2021). We calculate the growth rates of investment costs using the projected costs in NREL (2021).

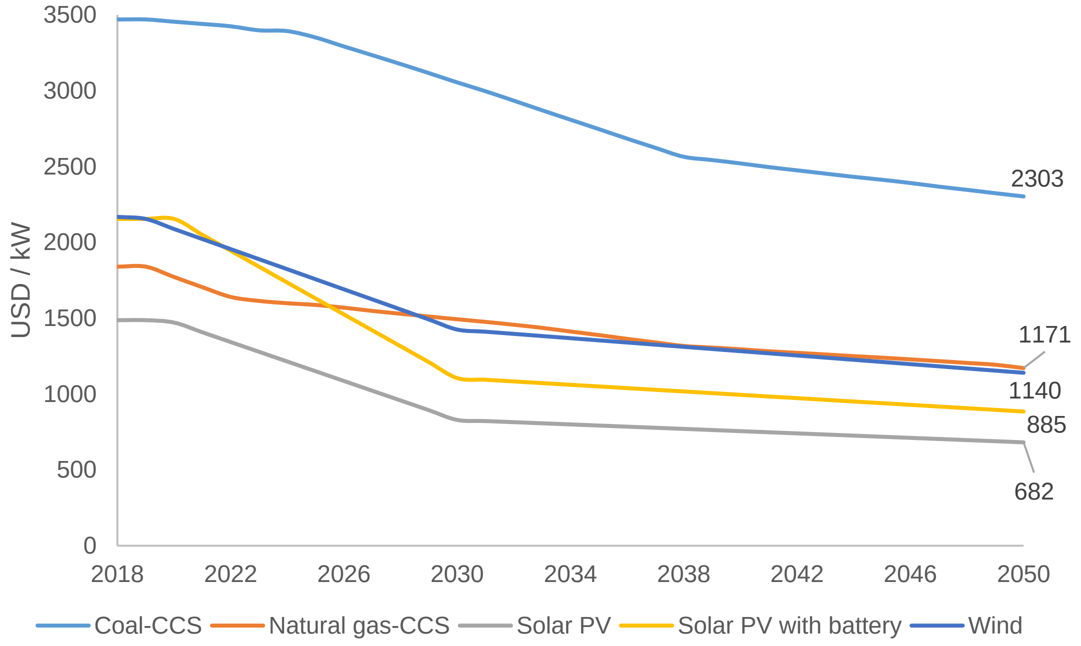

Figure S1. Investment cost trajectories of emerging generation technologies, 2018 – 2050 [Related to STAR Methods]

In this study, we consider the improvement in thermal efficiency for conventional generation technologies consuming coal and natural gas (Figure S2). In the long-run, we assume that the energy efficiency of coal-fired power plants in ASEAN can achieve the average efficiency level given to China and India (i.e., 45 percent) (IEA, 2020c), as what has been implemented in Huber, Roger, and Hamacher (2015). Similarly, the energy efficiency of natural gas power plants in ASEAN can be improved to the level of China (i.e., 58 percent) (IEA, 2020c). This also implies improvements in energy efficiency for coal with CCS and natural gas with CCS.

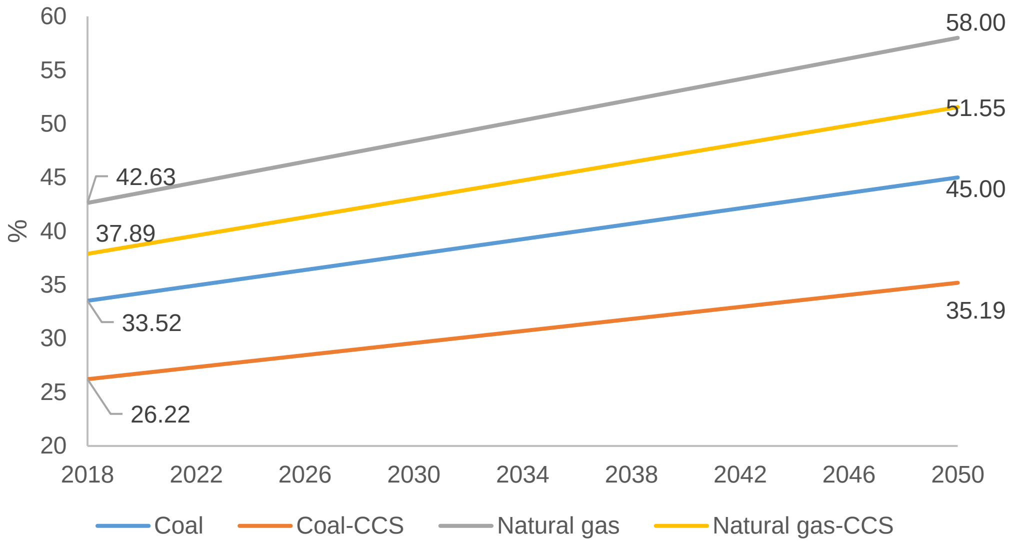

Figure S2. Efficiency trajectories of selected power generation technologies, 2018 – 2050 [Related to STAR Methods]

Table S2 presents the installed capacity by generation technology in 2018. For all countries, the initial capacity of CCS technologies is zero. The capacity data for hydro, geothermal, solar PV, wind and biomass are obtained from IRENA Renewable Capacity Statistics (IRENA, 2021a). For Indonesia, the data on non-renewable generation capacity (except for those with CCS) are based on Indonesia's official energy outlook (National Energy Council of Indonesia, 2019), and for Singapore, official data are used as well (EMA, 2021). For all other countries, data from IRENA Statistical Profiles (IRENA, 2021b) and plant-level microdata provided (Enerdata, 2021) are used.

Table S2. Installed capacity in ASEAN by power technology in 2018 (unit: MW) [Related to STAR Methods]

Country	Oil	Coal	Natural gas	Hydro	Geothermal	Solar PV	Wind	Biomass
Brunei	14	0	992	0	0	1.2	0	0
Cambodia	281	537	0	1,330	0	28.8	0.25	51
Indonesia	4,515	32,250	18,705	5,772	1,948	69.1	143.5	1,875
Lao PDR	0	1,878	0	5,256	0	21.6	0	40
Malaysia	1,347	10,102	14,270	6,165	0	536	0	839
Myanmar	120	173	2,631	3,304	0	47.5	0.01	5
Philippines	4,292	8,844	3,453	3,701	1,944	896	427	258
Singapore	807	1,748	10,681	0	0	160	0	257
Thailand	34	6,668	33,339	3,667	0.3	2,967	1,103	4,196
Vietnam	1,529	15,471	9,734	17,989	0	105	2,367	380
**ASEAN**	**12,938**	**77,671**	**93,804**	**47,185**	**3,893**	**4,833**	**1,910**	**7,900**
Share (%)	5.17	31.05	37.5	18.86	1.56	1.93	0.76	3.16

Given countries' commitment regarding coal power (UNFCCC, 2021), we have introduced several assumptions regarding the capacity expansion of coal and natural gas. For Malaysia

and Philippines, total capacity of coal without CCS before 2030 in the model is capped by the existing capacity plus the capacity of projects that are under construction, i.e., 14 GW for Malaysia and 12 GW for Philippines, as presented in the IEA study (IEA, 2020b). We further assume that for selected countries, by 2025, new capacity of natural gas without CCS in the model is equal to or larger than the capacity of under-construction projects with a commissioning year before 2025, i.e., 24.3 GW for Indonesia, 17.6 GW for Malaysia, 4.4 GW for Myanmar, 37.4 GW for Thailand and 16.1 GW for Vietnam (Enerdata, 2021).

To estimate the average age of plants, we calculate the difference between the commissioning year and 2018 for all ASEAN power plants covered in Enerdata's micro dataset (Enerdata, 2021). With the information of lifetime by generation technology, we estimate the remaining lifetime by generation technology by country, as shown in Table S3. For coal and natural gas, we distinguish between old and young plants, based on the distribution of plant-level remaining lifetime (Table S4). For those technologies that are not applicable (e.g., hydropower in Singapore) or did not exist in 2018 (e.g., generation technologies with CCS), the remaining lifetime is set to 0.

Table S3. Remaining lifetime of selected technologies in 2018 (unit: year) [Related to STAR Methods]

Country	Oil	Hydro	Geothermal	Solar PV	Wind	Biomass
Brunei	16	0	0	17	0	0
Cambodia	14	44	0	19	20	9
Indonesia	16	27	21	18	20	16
Lao PDR	0	43	0	20	0	15
Malaysia	9	33	0	20	0	15
Myanmar	15	37	0	20	20	19
Philippines	19	31	11	18	15	16
Singapore	16	0	0	17	0	11
Thailand	15	27	1	15	17	11
Vietnam	15	43	0	20	17	20

Table S4. Remaining lifetime of coal and natural gas power plants in 2018 (unit: year) [Related to STAR Methods]

Country	Coal1	Coal2	Natural gas1	Natural gas2
Brunei	0	0	13 (42.2%)	23 (57.8%)
Cambodia	37 (100%)	0	0	0
Indonesia	17 (51%)	33 (49%)	12 (53.7%)	25 (46.3%)
Lao PDR	37 (100%)	0	0	0
Malaysia	12 (53.5%)	28 (46.5%)	12 (61.4%)	23 (38.6%)
Myanmar	39 (100%)	0	12 (29.3%)	26 (70.7%)
Philippines	12 (43.4%)	36 (56.6%)	12 (41.2%)	21 (58.8%)
Singapore	36 (100%)	0	12 (42.2%)	23 (57.8%)
Thailand	7 (67%)	27 (33%)	12 (43.8%)	25 (56.2%)
Vietnam	9 (6.5%)	36 (93.5%)	12 (41.8%)	21 (58.2%)

Note: Percentage number in brackets indicates the capacity share of the technology in 2018.

For each country, we estimate capacity factor by generation technology. Table S5 below summarizes the estimates. The capacity factors of oil, coal and natural gas are based on the installed capacity we collected and the actual electricity production provided by the IEA (IEA, 2021c). Due to the issue of missing data, we use ASEAN-wide average coal capacity factor for Myanmar. For natural gas, we assume that if one country has installed capacity of natural gas in 2018, a minimum running requirement, ranging from 100% of regular capacity factor in 2018 to 75% in 2030 and 50% after 2035, is assigned in modelling for those existing natural gas plants through remaining lifetime.

For hydropower, geothermal and solar PV, capacity factors are obtained from the IRENA Statistical Profiles (IRENA, 2021b), except for Lao PDR and Malaysia in which the solar PV capacity factors from the World Bank are used (World Bank, 2021). The wind power capacity factor for Lao PDR is based on the assessment by Lee, Grue, and Rosenlieb (2018). For Malaysia, we estimate the wind power capacity factor using NREL's RE Explorer tool (NREL, 2022).[5] For Myanmar, the wind power capacity factor is calculated using the data in ACE (2019b). For the rest countries, similarly, the wind power capacity factor is provided by IRENA (2021b). Due to data availability, the representative biomass capacity factor in NREL (2021) is used in this study. According to NREL (2019), the technology with CCS has a capacity factor that is identical to that technology without CCS.

Table S5. Capacity factors by power technology in 2018 (%) [Related to STAR Methods]

Country	Oil	Coal	Natural gas	Hydro	Geothermal	Solar	Wind	Biomass	Coal-CCS	Natural gas-CCS	Biomass-CCS
Brunei	39.41	N.A.	48.84	N.A.	N.A.	15.00	N.A.	N.A.	N.A.	48.84	N.A.
Cambodia	12.16	64.97	N.A.	41.00	N.A.	17.00	14.00	62.50	64.97	N.A.	62.50
Indonesia	37.62	56.64	36.26	45.50	78.00	19.86	16.00	62.50	56.64	36.26	62.50
Lao PDR	N.A.	73.06	N.A.	46.00	N.A.	16.13	14.8	62.50	73.06	N.A.	62.50
Malaysia	8.88	87.33	51.11	49.00	N.A.	15.58	5.97	62.50	87.33	51.11	62.50
Myanmar	7.34	67.19	38.17	43.00	N.A.	17.00	22.28	62.50	67.19	38.17	62.50
Philippines	8.44	67.03	70.53	33.00	62.00	16.00	31.00	62.50	67.03	70.53	62.50
Singapore	4.49	4.49	53.94	N.A.	N.A.	12.24	N.A.	62.50	4.49	53.94	62.50
Thailand	60.01	62.33	39.60	35.00	75.00	17.00	17.00	62.50	62.33	39.60	62.50
Vietnam	1.93	84.25	48.94	54.00	N.A.	14.81	15.00	62.50	84.25	48.94	62.50

Notes: (1) N.A. = not applicable. (2) Solar in this table refers to solar PV and solar PV with battery.

We further consider the improvement in capacity factor of solar PV in the long-run. We have run simulations using the PVGIS platform developed by the Joint Research Centre of the European Commission (European Commission, 2022). This platform can estimate yearly electricity output for a given solar PV capacity (e.g., 1 kWp) in a specific location, and various PV cell technologies (e.g., crystalline silicon, CIS and CdTe) and tracking options (e.g., optimal slope and azimuth) are available. This allows us to calculate the capacity factor.

For each country, we select multiple locations that have very high solar irradiance. For each selected location within that country, we run several simulations by changing PV cell technology and allowing optimal PV tracking. The maximum capacity factor among all

[5] In NREL's RE Explorer tool, the Technical Potential module provides an assessment of electricity generation and nameplate capacity at country level for wind power in Malaysia, which allows us to calculate the capacity factor.

simulations is viewed as the country's long-run capacity factor. We apply such estimation procedures to all countries except for the Philippines due to data availability. In this study, we assume an annual growth of 1 percent in capacity factor for the Philippines. Figure S3 below presents the trajectories of solar PV capacity factors in ASEAN.

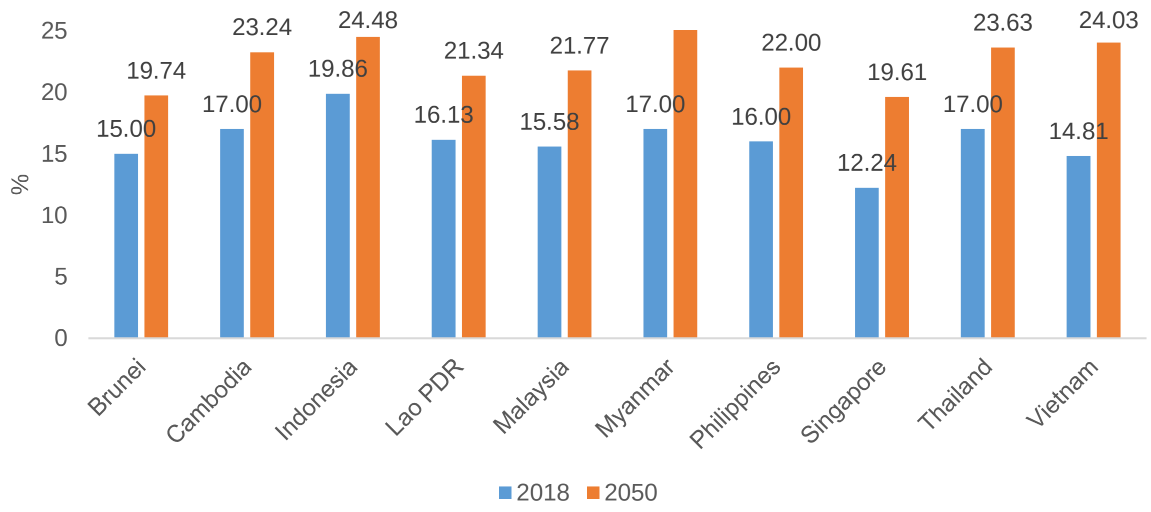

Figure S3. Trajectories of solar capacity factors (%) [Related to STAR Methods]

Fuel Prices

In this study, we consider the price dynamics of four fuel types over the entire period under research, namely, residual fuel oil, coal, natural gas and biomass. Figure S4 below shows the energy price trajectories. All fuel prices are expressed in USD per MWh, based on the conversion factors from BP (2021). Prior to the year 2020, the residual fuel oil of Thailand, obtained from the IEA Energy Prices dataset (IEA, 2021b), is used to represent the ASEAN price. From 2020 onwards, we derive residual fuel oil prices across modelling years using the oil price growth rates from the World Bank for 2018 – 2024 (World Bank, 2023), and the projected growth rates of residual fuel oil prices in the U.S. power sector for 2025 and onwards (EIA, 2023).

The historical prices of coal and natural gas are in accordance with the data from the World Bank Commodities Price Forecast (World Bank, 2023). Australia’s coal price (6000 kcal/kg) is used as the representative price for the Asia-Pacific region. The liquefied natural gas price of Japan hub is considered to be the representative natural gas price in Asia. Coal price projections are taken from the McCloskey Coal Price Outlook (OPIS, 2023). For natural gas price projections, we apply the price growth rates from the World Bank to the years prior to 2025 (World Bank, 2023), and price growth rates from the IEA Global Energy and Climate Model to the period 2025 – 2050 (IEA, 2022a). As shown in Panel A, natural gas prices are significantly higher than coal prices in ASEAN.

For biomass, we first estimate the price in 2018. In the literature, the average price of biomass materials in ASEAN in 20 USD / tonne (ACE, 2019a). In this study, we consider 5 types of biomass materials, i.e., forest residues, wood waste, agricultural residues, energy crops and landfill gas (IRENA, 2012). We assume the same price for those materials (in terms of USD / tonne). As the heat value varies across different biomass materials, the price expressed in

energy unit differs (IRENA, 2012). We use the average price in USD / MWh over all 5 types of biomass materials as the biomass price in ASEAN, i.e., 5.03 USD / MWh in 2018. From 2018 onwards, we derive the biomass price using the projected growth rates of biomass price developed by Paardekooper et al. (2018). Panel B of the figure below depicts the dynamics of biomass prices, which shows an increasing trend to 2050. The range of biomass prices in this study (or 1.47 – 1.77 USD / MMBtu) falls within the price range (1.3 – 3.5 USD / MMBtu) for ASEAN (Handayani et al., 2022).

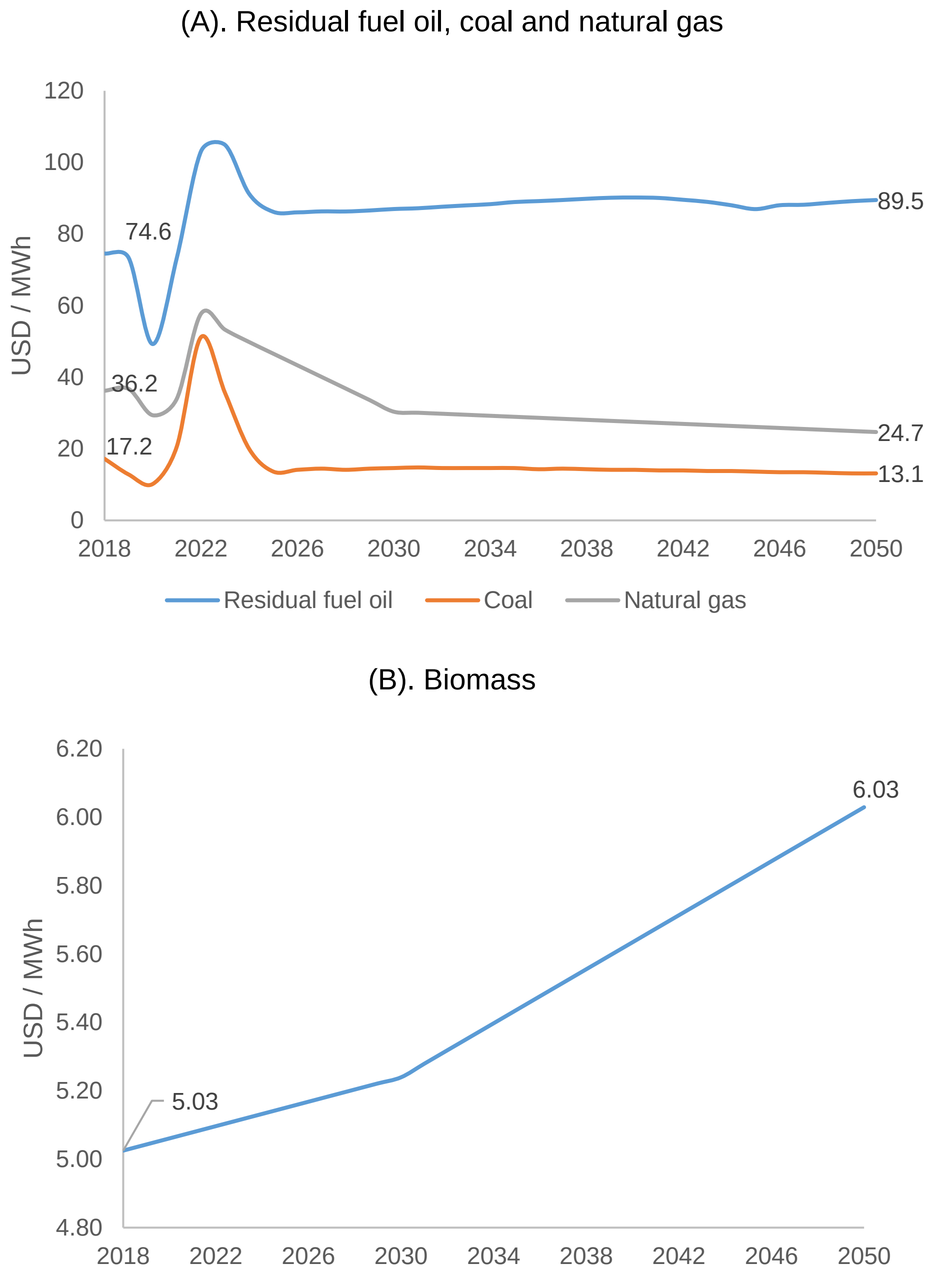

Figure S4. Trajectories of fuel prices, 2018 – 2050 [Related to STAR Methods]

Renewable Energy Resources

Table S6 below summarizes the renewable potentials by source and country. For hydro, solar, and wind, we explicitly distinguish between high and moderate levels of potential. In the table

below, the columns named “high” contain potentials that are not smaller than those in the “moderate” columns. The moderate renewable potentials represent those that are more likely to be achieved or derived based on policy targets.

The deployment of dams may lead to negative externalities to riverine ecosystems, fisheries and local communities (Siala, Chowdhury, Dang, & Galelli, 2021). Thus, the high hydro potentials in ASEAN, in particular, in the Greater Mekong region, may not be fully utilized. In this study, we estimate the moderate level of hydro potentials. For the Philippines, the government has set a hydropower target of 5349 MW by 2030 (Asian Development Bank, 2018b). A linear trend between the capacity in 2018 and the target in 2030 is interpolated. We assume the same trend from 2030 onwards and derive the 2050 value of 8.2 GW, which serves as the achievable upper limit for hydro resources in the Philippines. For the other countries, we estimate the upper limits of hydro capacity given historical trends. We calculate the 5-year moving average hydro capacity for each country, in order to obtain smooth trends over time. This is done by using the historical data on hydro capacity from IRENA (2021a). For each country, a linear line is fitted using the 5-year moving average and time variable for the period 2010 – 2020, and the 2050 value is derived based on such a linear trend. For Vietnam, the interpolated hydro capacity exceeds the high level of hydro resource (i.e., 35 GW), and thus 35 GW is used as the moderate hydro capacity as well.

For solar and wind, the high resource potentials are in accordance with technical assessments from ACE (2019b); NREL (2022). The moderate potentials include smaller estimates from the literature as well as those that are released by official institutions. In cases where the technical assessment (ACE, 2019b; NREL, 2022) gives potentials that are smaller than official releases, data from the latter are used as high potentials (e.g., solar in Singapore and wind in Indonesia). Similar to the hydro resource, the government of the Philippines has set its targets for wind power by 2030, i.e., 2.35 GW (Asian Development Bank, 2018b). We first fit a linear line connecting the capacity in 2018 and the targeted capacity in 2030 and interpolate the capacity in 2050 using such a linear trend, which is viewed as the moderate wind potential for the Philippines. Using the same linear interpolation approach, we estimate the wind power potential for Vietnam (Baker & McKenzie, 2021). The Vietnamese government expects a fast growth of solar capacity over 2030 – 2045 (Baker & McKenzie, 2021), and we estimate the capacity in 2050 using linear interpolation.

For solar PV and solar PV with battery, we then use an S curve approach to derive upper limit of capacity expansion of country i in year t, $USolar_{i\,t}$, as shown below

$$USolar_{i\,t} = \frac{Max\_solar_i}{1 + e^{p(t-2035)}}$$

where Max_solar_i is the solar resource potential of country i, p is the logistic growth rate.

We assume that capacity can achieve up to 50 percent of solar resource potential in 2035 (i.e., using 2035 as the curve’s midpoint). p is estimated using the historical capacity data for 2020 (IRENA, 2021a).

Table S6. Renewable energy resources by ASEAN country (unit: GW) [Related to STAR Methods]

Country	Hydro		Geothermal	Solar		Wind		Biomass
	High	Moderate		High	Moderate	High	Moderate	
Brunei	0	0	0	16.0	2.5	0	0	0
Cambodia	10.0	5.5	0	3198.0	8.1	69.0	65.0	2.15

Indonesia	94.0	13.8	29.0	1052.0	208.0	61.0	61.0	33.00
Lao PDR	26.0	20.3	0	1278.0	8.8	13.0	3.5	1.20
Malaysia	35.0	20.6	0	1965.0	6.5	2.0	2.0	3.67
Myanmar	19.6	9.0	0	7717.0	30.0	482.0	6.97	0.99
Philippines	13.1	8.2	4.5	1910.0	45	217.0	5.54	4.45
Singapore	0	0	0	8.6	8.6	0	0	0.26
Thailand	6.4	4.1	0.0003	10538.0	22.8	239	17.00	7.00
Vietnam	35.0	35.0	0	2847.0	71.7	311	27.75	1.00

Source: Hydro (high): (Asian Development Bank, 2018a; Government of Indonesia, 2018; Government of Myanmar, 2021; Handayani et al., 2022; Huber, Roger, & Hamacher, 2015; Paltsev et al., 2018); Geothermal: (Government of Indonesia, 2018; Paltsev et al., 2018); Solar (high): (ACE, 2019b; NREL, 2022); Solar (moderate): (Ahmad, Ab Kadir, & Shafie, 2011; Asian Development Bank, 2019; Baker & McKenzie, 2021; DOE of the Philippines, 2021; Government of Indonesia, 2018; Government of Myanmar, 2021; Huber, Roger, & Hamacher, 2015; IRENA, 2021a; NCCS, 2020); Wind (high): (ACE, 2019b; NREL, 2022) ; Wind (moderate): (Asian Development Bank, 2018a, 2018b, 2019; Baker & McKenzie, 2021; Government of Indonesia, 2018; Government of Myanmar, 2021; IRENA, 2017a); Biomass: (Government of Indonesia, 2018; Handayani et al., 2022; Huber, Roger, & Hamacher, 2015; IRENA, 2017b; Tun, Juchelkova, Win, Thu, & Puchor, 2019).

Notes: (1) zero in this table means the renewable energy resource is not applicable or not considered in that country. (2) Due to data availability, geothermal resource in Thailand is assumed to remain unchanged from its installed capacity in 2018. This is in line with the geothermal resource given to Thailand in literature (nearly 0 GW) (Handayani et al., 2022; Huber, Roger, & Hamacher, 2015). Biomass resource in Singapore is set to its installed capacity in 2018 as well. Moderate wind resource in Malaysia is assumed to be identical to the high potential value.

Transmission Lines

In this study, two types of transmission technologies are included, i.e., HV onshore and HV offshore. The table below summarizes the characteristics of both transmission technologies (Stich & Massier, 2015).

Table S7. Characteristics of transmission technologies in ASEAN [Related to STAR Methods]

Transmission technology	Investment (USD / MW km)	Fixed O&M cost (USD / km a)	Variable cost (USD / MWh)	Efficiency (%)	Lifetime (years)
HV onshore	500	10	0	90	40
HV offshore	3500	70	0	90	40

Table S8 below presents the information on the edges that form the transmission network. This table contains the cross-border transmission lines and capacity that are currently operational as well as those that are under construction.

The data on transmission capacity and the related lines are primarily collected from IEA (2019a, 2019b). The transmission capacity between Indonesia and Singapore is taken from Chang, Lee, Ang, and Chua (2019). We calculate the length for the following transmission lines: Malaysia – Indonesia, Philippines – Malaysia, Malaysia – Brunei and Indonesia – Singapore. To do so, we first identify the connecting locations of each country pair, as indicated in the parentheses, based on the IEA Southeast Asia Energy Outlook (IEA, 2019b). Then we calculate the great circle distance between those two connecting locations for each

country pair. This draws on the approach proposed in the literature (Feenstra, Markusen, & Rose, 2001; Lugovoy, Gao, Gao, & Jiang, 2021). The data for all other transmission lines are taken from ERIA (2014).

Table S8. Transmission lines in ASEAN Power Grid [Related to STAR Methods]

Transmission line		Capacity (GW)	Length (km)	Type
Thailand - Lao PDR		6.77	213	HV onshore
Lao PDR - Vietnam		0.54	192	HV onshore
Thailand - Myanmar		14.86	450	HV onshore
Vietnam - Cambodia		0.20	88	HV onshore
Lao PDR - Cambodia		0.30	56	HV onshore
Thailand - Cambodia		2.32	290	HV onshore
Malaysia - Singapore		1.05	24	HV onshore
Malaysia - Indonesia	Malaysia (Peninsular) - Indonesia (Sumatra)	0.60	295	HV offshore
	Malaysia (Sarawak) - Indonesia (West Kalimantan)	0.23	300	HV onshore
Philippines (Palawan) – Malaysia (Sabah)		0.50	529	HV offshore
Malaysia (Sarawak) - Brunei		0.40	111	HV onshore
Thailand - Malaysia		0.78	132	HV onshore
Indonesia - Singapore	Indonesia (Batam) - Singapore	0.60	38	HV offshore
	Indonesia (Sumatra) - Singapore	0.60	84	HV offshore

To provide data inputs to URBS, we further calculate the investment cost (in the unit of USD / MW) and fixed O&M cost (in the unit of USD / MW a), taking into account transmission length and type of technology. The following calculation is used.

Let $AC^{i\,j}$ denote the adjusted investment cost of the transmission line connecting country i and j expressed in the unit of USD / MW. Between country i and j, there are n onshore lines and m offshore lines. Then $AC^{i\,j}$ is calculated as follows.

$$AC^{i\,j} = \frac{\sum_{k=1}^{n}(C^{on}Cap_k^{ij,on}L_k^{ij,on}) + \sum_{k=1}^{m}(C^{off}Cap_k^{ij,off}L_k^{ij,off})}{\sum_{k=1}^{n}Cap_k^{ij,on} + \sum_{k=1}^{m}Cap_k^{ij,off}}$$

where

$Cap^{ij,on}$ and $Cap^{ij,off}$ indicate the capacity of onshore line and offshore line, respectively.

$L^{ij,on}$ and $L^{ij,off}$ refer to the transmission length of onshore line and offshore line, respectively.

C^{on} and C^{off} are the investment cost per unit of capacity per kilometre for onshore line and offshore line, respectively.

Using the data in Table S7, we obtain the following adjusted investment cost and fixed O&M cost for each transmission line.

Table S9. Investment cost and fixed O&M cost of transmission lines in ASEAN [Related to STAR Methods]

Transmission line	Investment (USD / MW)	Fixed O&M cost (USD / MW a)
Thailand - Lao PDR	106,667	2,133
Lao PDR - Viet Nam	96,000	1,920

Thailand - Myanmar	225,000	4,500
Viet Nam - Cambodia	44,000	880
Lao PDR - Cambodia	28,000	560
Thailand - Cambodia	145,000	2,900
Malaysia - Singapore	12,000	240
Malaysia - Indonesia	787,959	15,759
Philippines - Malaysia	1,850,849	37,017
Malaysia - Brunei	55,361	1,107
Thailand - Malaysia	66,000	1,320
Singapore - Indonesia	213,273	4,265

Electricity Demand

In this study, we develop country-specific electricity demand projections based on ACE (2020); ERIA (2021). ACE's energy outlook projects ASEAN's total electricity demand until 2040 by incorporating ASEAN member states' major policy targets (known as ATS scenario) (ACE, 2020). Following Singapore's EV plan (Land Transport Authority, 2022), we assume that all gasoline vehicles will be replaced by EVs by final model year. At ASEAN level, adding the energy demand from gasoline vehicles to electricity demand under ATS gives an increase of 15.7 percent in total electricity demand in final year, as compared to that under BAU (ACE, 2020). We then apply such an increase to ASEAN's BAU electricity demand reported in ERIA (2021) for the year 2050, and interpolate the demand between 2018 and final model year. Based on country's share in total electricity demand under BAU (ERIA, 2021), we derive country-specific electricity demand. Our projection for the entire ASEAN in 2050 is close to that in IRENA (2022).

Emission Targets

All ASEAN member states have committed to reducing their emissions or emission intensity in order to achieve the targets set by the Paris Agreement. In this study, we focus on the countries' unconditional emission targets, i.e., the targets that are achievable based on domestic efforts and capabilities. Total emissions under unconditional NDC targets are collected from official releases by governments where available. The total emissions of Lao PDR, Malaysia, Myanmar and the Philippines are taken from Paltsev et al. (2018). We then estimate the emissions from power sector under unconditional NDC targets except for Myanmar in which this information is clearly indicated in official release. For countries where applicable, this is done by using the share of power sector in total emissions in 2018 (IEA, 2021a). Table S10 presents national emissions and the emissions from power sector under unconditional NDC targets.

Table S10. 2030 emission targets under unconditional NDC by ASEAN country (unit: $MtCO_2$) [Related to STAR Methods]

Country	National emissions	Emissions from power sector
Brunei	23.6 (Government of Brunei Darussalam, 2020)	10
Cambodia	52.3 (Kingdom of Cambodia, 2020)	16
Indonesia	1817 (Government of Indonesia, 2021)	723
Lao PDR	22.5 (Paltsev et al., 2018)	18
Malaysia	539.8 (Paltsev et al., 2018)	266

Myanmar	72.8 (Paltsev et al., 2018)	34 (Government of Myanmar, 2021)
Philippines	293.1 (Paltsev et al., 2018)	154
Singapore	65 (Government of Singapore, 2020)	28
Thailand	444 (Government of Thailand, 2020)	161
Vietnam	844.4 (Government of Viet Nam, 2020)	407

Scenario Development

Three scenarios have been implemented in this study (Table S11). To design these scenarios, we focus on three key areas, i.e., cross-border transmission, resources and decarbonization.

In scenarios with the cross-border transmission, net electricity imports can contribute to a maximum of 30% of Singapore's domestic demand by 2035 and up to 60% by 2050. The high electricity import share is based on the assessment by the Government of Singapore (EMA, 2022). We apply Singapore's import constraints to Brunei. For all other countries, net electricity imports can account for up to 20% of domestic demand by 2035, which is based on the share of electricity imports of Cambodia in 2018 (IEA, 2021c), and up to 30% by 2050. For Cambodia and Thailand, which have been major electricity importers, we assume that net electricity imports contribute to at least 10% of domestic demand each year, which is based on the import share of Thailand in 2018 (IEA, 2021c). For Vietnam, net electricity imports will contribute to at least 8% from 2030 and onwards (Vietnam Electricity, 2020). In addition, we apply various export limits (See Table S11).

In the area of resources, we distinguish between the moderate and high renewable potentials as presented in Table S6. In the area of decarbonization, the primary setting, i.e., moderate emissions targets, is the unconditional emission targets set by ASEAN countries' NDCs. We further assume emissions can be reduced by half from the 2030 level. This is based on Singapore's Long-Term Low-Emissions Development Strategy (LEDS) (NCCS, 2021), and in line with the setting in IEA's sustainable development scenario (IEA, 2022b). Between 2030 and the final model year, we assume linear declining trends in emissions for all countries. Further, in the ambitious mitigation scenario, we consider net-zero emissions for the power sector by 2050.

Table S11. Scenario description **[Related to Table 1]**

Scenario	Description
S1 (Autarky)	• No cross-border transmission allowed<br>• Moderate renewable resources<br>• Moderate emissions targets (2030 NDCs and halve emissions by 2050)
S2 (Grid Integration)	• Planned transmission network and capacity expansion allowed<br>➢ *Net electricity imports can provide up to 30% of Singapore's demand by 2035, and up to 60% by 2050*<br>➢ *Brunei relies on domestic generation*<br>➢ *For all other countries, net electricity imports can provide up to 20% of domestic demand by 2035, and up to 30% by 2050*<br>➢ *Minimum net electricity imports: 10% for Cambodia and Thailand in each year, 30% for Singapore from 2040 and onwards, and 8% for Vietnam from 2030 and onwards*<br>➢ *No electricity exports from Brunei, Cambodia, Thailand and Singapore. Malaysia can export electricity from 2030 and onwards*<br>• Moderate renewable resources

	• Moderate emissions targets (2030 NDCs and halve emissions by 2050)
S3 (Ambitious Mitigation)	• Planned transmission network and capacity expansion allowed<br>➢ *Brunei has the same imports constraints with Singapore*<br>➢ *All other settings are same with S2 (Grid Integration).*<br>• Resource exploration with higher hydro, solar and wind potentials<br>• Low emissions targets (2030 NDCs and zero emissions by 2050)

Mathematical Model

The notation of the model is introduced below:

Nomenclature

Indices:

V	The set of regions $v \in V$.
E	Set of plausible transmission lines from region v_1 to v_2, $E = \{(v_1, v_2) \mid v_1, v_2 \in V\}$
Y	Set of modeled years $y \in Y, Y := \{y_1, \ldots, y_{\|Y\|}\}$
G	The set of technologies $g \in G$
P	The set of available generation technologies in different regions, $P = \{(g, v) \mid g \in G, v \in V\}$
C	The set of commodities, including the set of fuels C^F and CO_2 emissions, i.e., $C = C^F \cup \{CO_2\}$
$\mathcal{A}$	The set of all assets, including all available generation facilities and all transmission lines, i.e., $\mathcal{A} = P \cup E$
$Y(y,a)$	The set of modelled years that an asset $a \in \mathcal{A}$ remains operational if it is installed in year $y \in Y$; we further define $Y(0,a)$ as the set of operational years of the initially installed asset a.
S	The set of technology groups; the members of a group $s \in S$ share the same capacity limits
P_s	The set of generation technologies (g,v) in group $s \in S$

Parameters:

D_{yv}	Projected electricity demand for region $v \in V$ in year $y \in Y$
$K_{ya}^{Capex}, K_{ya}^{Fix}$	The unit CAPEX cost and FOM cost (USD per MW) of an asset $a \in P \cup E$ in year y, respectively
K_{ya}^{Var}, K_{ya}^{F}	The unit variable cost and unit fuel cost (USD per MWh) of an asset $a \in P \cup E$ in year y, respectively
$\widehat{\kappa}_{0a}$	Installed capacity of asset a in the initial year.
$\overline{\widehat{\kappa}}_{0a}$	The upper bound of adding new capacity of asset a in year y.
$\underline{\kappa}_{ya}, \overline{\kappa}_{ya}$	The upper bound of asset a in year y
$\underline{\kappa}_{ys}, \overline{\kappa}_{ys}$	The upper bound of technology group s in year y
n_a	The lifetime of an asset $a \in P \cup E$.

$l_{v_1v_2}$	The transmission efficiency factor.
$\underline{\lambda}_{yv}, \overline{\lambda}_{yv}$	The minimum and maximum electricity net import for region v in year y.
$\underline{\eta}_{ya}, \overline{\eta}_{ya}$	The minimum and maximum capacity factor for an infrastructure asset a in year y
T	The number of hours per year (8760 hours)
F_{ygvc}	The input (or output) ratio of commodity $c \in C$, in each asset (g, v)
$\overline{\rho}_{yvc}$	The upper bound of commodity $c \in C$ in region v in year y
Decision Variables	
ζ	Total cost
$\kappa_{ya}, \hat{\kappa}_{ya}$	Total available capacity and newly installed capacity of an asset $a \in P \cup E$ in year y, respectively
τ_{ya}	The electricity throughput of an asset $a \in P \cup E$ year y. τ_{ya} represents the generated electricity for $a \in P$, and the transmitted electricity in transmission line $a \in E$.
ρ_{ygvc}	The input (or output) of commodity $c \in C$ of process (g, v) in year y

The model minimizes the total system costs, including the net present value (NPV) of total capital expenditure (CAPEX) costs ζ^{Capex} of newly built generation plants and transmission capacities, the fixed O&M (FOM) costs ζ^{FOM} of these assets, the variable costs ζ^{Var} and the fuel costs ζ^{F} for electricity generation. The transmission grids are modeled as directed arcs that connect the nodes, with two separate directions for each transmission line. A coefficient of $1/2$ is used to avoid overcounting the CAPEX costs and fixed costs of a transmission line. The total costs are calculated as follows:

$$\min \zeta = \sum_{y \in Y} \sum_{a \in P} \zeta_{ya}^{Capex} + \sum_{y \in Y} f_y(\omega_y) \cdot \left(\sum_{a \in P} (\zeta_{ya}^{FOM} + \zeta_{ya}^{Var}) + \sum_{c \in C^F, v \in V} \zeta_{yrc}^{F} \right) + \frac{1}{2} \sum_{y \in Y, a \in E} \left(\zeta_{ya}^{Capex} + f_y(\omega_y) \zeta_{ya}^{FOM} \right) \quad (1)$$

where $f_y(\omega_y)$ is the NPV factor for the annuitized cost in year y with a weight ω_y. The weight of a modeled year y_n equals the length of the time interval between year y_n and the next modeled year y_{n+1}, i.e., $\omega_{y_n} = y_{n+1} - y_n$. Therefore, the NPV factor calculated as $f_y(\omega) = (1+j)^{1-(y-y_1)} \frac{1-(1+j)^{-\omega}}{j}$, where j is the discount rate and y_1 represents the initial year. Essentially, the function $f_y(\omega)$ gives the NPV factor of annuities that start in year y and repeat ω_y times. It implies that the same cost occurs in each year represented by the modeled year y.

The CAPEX of the newly installed capacity of asset $a \in P \cup E$ in year y is computed as the sum of annuities to distribute equally over the lifetime of the asset. Only the payments that fall within the planning horizon are counted and discounted to the commission year. This is a common practice in many energy system models ((Howells et al., 2011), LEAP, TIMES) to ensure the salvage value at the end of the planning horizon is properly assessed. The cost is calculated as follows:

$$\zeta_{ya}^{Capex} = f_y(n_{ya}) f_a K_{ya}^{Capex} \hat{\kappa}_{ya} \tag{2}$$

where $n_{ya} = \min\left\{n_a, y_{|Y|} - y\right\})$ denotes the number of payments that fall in the planning horizon and $f_a = \dfrac{(1+i)^{n_a} \cdot i}{(1+i)^{n_a} - 1}$ is the capital recovery factor with interest rate i and asset lifetime n_a.

The rest types of costs are calculated as:

$$\zeta_{ya}^{FOM} = K_{ya}^{FOM} \kappa_{ya} \tag{3}$$

$$\zeta_{ya}^{Var} = K_{ya}^{Var} \tau_{ya} \tag{4}$$

$$\zeta_{yrc}^{F} = \sum_{(g,r)\in P} K_{yrc}^{F} \rho_{ygrc} \tag{5}$$

The linear programming model is subject to a set of constraints on the properties of the power system, for electricity dispatch, generation capacity expansion and transmission. The cost minimization problem is formulated as follows.

$$\min \zeta \tag{6}$$

$$\text{s.t.} \sum_{(g,v)\in P} \tau_{ygv} + \sum_{v':(v',v)\in E} l_{v'v} \tau_{yv'v} - \sum_{v':(v,v')\in E} \tau_{yvv'} \geq D_{yv}, \forall y \in Y, v \in V \tag{7}$$

$$\underline{\lambda}_{yv} \leq \sum_{v':(v',v)\in E} l_{v'v} \tau_{yv'v} - \sum_{v':(v,v')\in E} \tau_{yvv'} \leq \overline{\lambda}_{yv}, y \in Y, v \in V \tag{8}$$

$$T \underline{\eta}_{ya} \kappa_{ya} \leq \tau_{ya} \leq T \overline{\eta}_{ya} \kappa_{ya}, \forall y \in Y, a \in A \tag{9}$$

$$\kappa_{ya} = \sum_{y'\in Y(y,a)} \hat{\kappa}_{y'a} + \mathbf{I}_{Y(0,a)}(y) \kappa_{0a}, \forall y \in Y, a \in A \tag{10}$$

$$\underline{\kappa}_{ya} \leq \kappa_{ya} \leq \overline{\kappa}_{ya}, \forall y \in Y, a \in A \tag{11}$$

$$\hat{\kappa}_{ya} \leq \hat{\overline{\kappa}}_{ya}, \forall y \in Y, a \in A \tag{12}$$

$$\kappa_{y,v_1,v_2} = \kappa_{y,v_2,v_1}, \forall y \in Y, (v_1, v_2) \in E \tag{13}$$

$$\rho_{ygvc} = F_{ygc} \tau_{ygv}, \forall y \in Y, (g,v) \in P, c \in C \tag{14}$$

$$\sum_{g \in G} \rho_{ygvc} \le \bar{\rho}_{yvc}, \forall y \in Y, (g,v) \in P, c \in C \tag{15}$$

$$\underline{\kappa}_{ys} \le \sum_{a \in P_s} k_{ya} \le \bar{\kappa}_{ys}, \forall y \in Y, s \in S \tag{16}$$

$$\boldsymbol{\zeta}, \boldsymbol{\kappa}, \hat{\boldsymbol{\kappa}}, \boldsymbol{\tau} \ge 0, \boldsymbol{\rho} \in \mathbb{R} \tag{17}$$

Constraint (7) maintains the electricity balance for each node. It ensures that domestic electricity generation in each country, as well as that net imports from other countries, satisfy projected demand D_{yr} in each country and year. The imports from other countries are discounted with the transmission efficiency factor $l_{v_1 v_2}$, due to the electricity loss on transmission lines. For the transmission of electricity over long distances across multiple countries, the power loss will multiply. As the core of the network model, the constraint allows for a detailed analysis on the transmission lines.

Constraints (8) capture each region's preferences regarding electricity trade, which provide boundaries on the annual net import in both directions for each country and year. A value of $\bar{\lambda}_{yv} = 0$ means that the region does not import electricity for its domestic consumption, while $\underline{\lambda}_{yv} = 0$ means that the region is not willing to export electricity. The constraints also provide the flexibility to determine a country's level of self-sufficiency by setting the net import upper bound $\bar{\lambda}_{yv}$ at the maximum desired level of trade, for example a proportion of the annual domestic demand. This prevents a country from becoming totally dependent on imports and, in the case of ASEAN, ensures electricity security for each member state.

To our knowledge, only (Stich & Massier, 2015) has included a similar constraint of overall net imports limit, but no details have been disclosed. The current constraint is unique in that it provides a more comprehensive and nuanced understanding of power trading preferences and strategies, which is lacking in many studies focusing on transmission lines in China and Europe, e.g. (Siala, de la Rúa, Lechón, & Hamacher, 2019; H. Wang et al., 2020).

The general rules for dispatch and capacity expansion are similar for both generation infrastructure P and transmission lines E, and they are summarized by constraints (9-11). Constraint (9) limits the amount of electricity generated (or transmitted) by the total operational capacity of the corresponding asset and the annual capacity factor. In terms of dispatchable infrastructure like fossil fuel power and transmission lines, the capacity factor represents the percentage of available working time per year, and for intermittent renewable resources, it reflects the average availability. Besides, the maximum capacity factor for transmission lines helps to model unidirectional electricity trading. The minimum capacity factor, on the other hand, characterise the minimum running requirements of the generation facilities. The available capacity is calculated in Constraint (10), where $Y(y,a) = \{y' \mid y' \in Y, y' + n_a \ge y\}$ is the set of building years of facility a that are operational in year y. In the second term of the right-hand side, we let $Y(0,a)$ denote the set of modeled years when the initially installed facility a is operational, such that the binary indicator function $\mathbf{I}_{Y(0,a)}(y)$ helps to include the

initial capacity κ_{0a} in the total available capacity if $y \in Y(0,a)$. Constraint (11) is a time-dependent upper bound on total available capacity. This constraint is typically necessary for renewable resources such as solar, hydro, and wind, due to their limited potential.

In addition, Constraint (12) gives an upper-bound on the maximum expansion rate for each type of generation infrastructure. Such bounds reflect conceivable policies, such as the restriction on coal power. Constraint (13) ensures that two directions of a transmission line have equal capacities.

The other commodity inputs and outputs of the electricity generation process $(g, v) \in P$ per year are characterized by Constraint (14). The total amount of commodity c associated with a generation facility is pro-rated to the electricity generation with an input (or output) ratio. For fuels $c \in C^F$, $\frac{1}{F_{ygc}}$ represents the efficiency of the generation technology when fuel c is fed. For CO_2 emissions, F_{ygc} represents the emission intensity. As expressed in Constraint (17), the non-negative restriction does not apply to these variables for fuels and emissions, which enables the modelling of negative emission technologies, such as Biomass-CCS. Constraint (15) limits the maximum allowance for each commodity per region and year. For fuels like biomass, annual consumption can be restricted by the projected availability. This constraint also restricts the annual CO_2 emissions for each node (country) according to the NDC goals. Unlike many other studies, this model does not include a constraint for CO_2 emission targets for ASEAN as a whole. Instead, setting the emission targets based on the countries' policies is more realistic.

Finally, Constraints (16-17) set capacity and generation limits for groups of generation infrastructure. The constraint (16) sets a group capacity limits, as one form of energy can be harnessed by multiple competing technologies, for example Solar and Solar with battery storage. These technologies in one group may share an aggregated target (lower bound) as well.

Comparison with Prior Studies

In this section, we review prior studies and identify the research gaps. Initial works focused on providing economical solutions to meet rapidly growing electricity demand, especially through power grid connections in the Great Mekong Subregion (Siala et al., 2021; Watcharejyothin & Shrestha, 2009; Yu, Bowen, Sparrow, Siriariyaporn, & Yu, 2005). However, with the on-going realization of the ASEAN Power Grid and deepening regional energy integration, recent attractions shifted to interregional transmission and trade across ten member states of ASEAN, in the context of deep decarbonization. Several studies have assessed the masterplan of APG. The study by Ahmed, et al. examined HVAC versus HVDC technologies but did not provide detailed analysis on the power sector's generation mix (Ahmed, Mekhilef, Shah, & Mithulananthan, 2017).

In particular, the literature on ASEAN's net-zero energy transition remains sketchy. Table S12 summarizes the key studies of net-zero energy transition on ASEAN and major regions (e.g., the global economy, China and the U.S.). We highlight the research gaps in the literature in the column "Research Gap / Critical Remarks".

As shown in Table S12, those studies focusing on the global economy have looked at pathways of emissions or temperature, instead of the transition trajectories of power sector, and ASEAN-specific results are rare in such studies. In the studies for individual economies,

e.g., China and the U.S., the model structure and analysis are specific to the countries under research. For those studies on ASEAN, several studies have focused on a single milestone year (e.g., 2050), instead of developing pathways through 2050. A key research gap is that cross-border transmission is not explicitly modelled. In addition, in some of the ASEAN studies, the transition pathways may not be the cost-optimal ones.

This study seeks to fill critical knowledge gaps by: 1) thoroughly assessing detailed pathways for ASEAN's power sector through 2050, 2) explicitly modelling both the expansion of the ASEAN Power Grid infrastructure and incorporating country-specific NDC targets and net-zero targets, and 3) analyzing multiple scenarios to evaluate trade-offs and system transitions.

Table S12. Literature Review on Net-Zero Energy Transition: Key Research Areas and Geographics [Related to STAR Methods]

Study Title	Authors (Year)	Research Area	Geographical Location	Findings	Research Gap/Critical Remarks
World Energy Outlook 2023	IEA (2023)	Energy systems modelling; energy projections	The whole world and major regions	Net-zero pathways for the global economy	Results under net-zero scenario are not available for Southeast Asia or individual ASEAN countries
Net-Zero Emissions Energy Systems	Davis et al. (2018)	Energy transition; technology assessment	World economy	The barriers and opportunities for net-zero energy systems	Technology-specific analysis; no country-level results
The Hydrogen Economy Can Reduce Costs of Climate Change Mitigation by up to 22%	Wolfram, Kyle, Fuhrman, O'Rourke, and McJeon (2024)	Integrated Assessment Models	World economy	Clean hydrogen can reduce global decarbonization costs by up to 22%	Only results for the global economy and the U.S.;<br>No results for power sector
Deep Mitigation of CO2 and Non-CO2 Greenhouse Gases toward 1.5 °C and 2 °C Futures	Ou et al. (2021)	Integrated Assessment Models	World economy	System-wide all GHG mitigation can affect the timing of net-zero	Analysis focusing on economy-wide emissions pathways, no results for power sector or investment;<br>No country-level results for ASEAN
Realization of Paris Agreement Pledges may Limit Warming just below 2 °C	Meinshausen et al. (2022)	Integrated Assessment Models	196 economies	Full implementation of climate pledges may limit warming just below 2 degrees Celsius	Analysis focusing on various pathways of emissions and temperature, no results on the energy transition pathways of power sector or demand sectors
Energy Systems in Scenarios at Net-Zero CO2 Emissions	DeAngelo et al. (2021)	Climate change scenarios	World economy and major regions	Renewables account for about 60% of primary energy, and electricity makes up about half of final energy	Results only available for major regions (e.g., the whole Asia);<br>Analysis based on comparisons of results from literature
Net-Zero Emission Targets for Major Emitting Countries Consistent with the Paris Agreement	van Soest, den Elzen, and van Vuuren (2021)	Integrated Assessment Models	World economy and major emitting countries	carbon storage and afforestation capacity, income, share of non-CO2 emissions, and transport sector affect the timing of emissions neutrality	For ASEAN, results only available for Indonesia;<br>Cross-border transmission is not incorporated;<br>No results for power sector pathways or investment
Mission Net-Zero America: The Nation-Building Path to a Prosperous, Net-Zero Emissions Economy	Jenkins, Mayfield, Larson, Pacala, and Greig (2021)	Energy system optimization model	USA	The deployment of solar and wind and the employment effects in the five distinct net-zero pathways for the U.S.	Analysis only for the U.S., no cross-border electricity transmission;<br>No results on the investment in infrastructure

Carbon-Neutral Pathways for the United States	Williams et al. (2021)	Energy systems modelling; partial equilibrium framework	USA	Multiple feasible net-zero pathways available to the US	Model and results are specific to the U.S.;<br>Only domestic transmission
The role of natural gas in reaching net-zero emissions in the electric sector	Bistline and Young (2022)	Energy system optimization model	USA	Gas-fired generation can lower the cost of net-zero transition	Analysis specific to the U.S.;<br>No cross-border transmission
Biomass Enables the Transition to a Carbon-Negative Power System across Western North America	Sanchez et al. (2015)	Energy systems modelling; power dispatch model	Western North America (11 western U.S. states, Northern Baja Mexico, and British Columbia and Alberta of Canada)	Bioenergy with CCS can enable a carbon-negative power system in western North America	Model settings and results are specific to the Western North America;<br>Countries' NDCs are not considered in scenarios
Accelerating the Energy Transition towards Photovoltaic and Wind in China	Y. Wang et al. (2023)	Optimization of PV and wind systems	China	Deployment strategies and welfare effects of solar and wind in China's net-zero pathways	Model and analysis specific to China;<br>Only domestic grids in the model; no cross-border transmission
Evaluating the Use of BECCS and Afforestation under China's Carbon-Neutral Target for 2060	Weng, Cai, and Wang (2021)	Computable general equilibrium (CGE) model of climate change	China	Deploying BECCS and afforestation could reduce the cost of net-zero emissions	Model and the social accounting matrix specific to China;<br>Power transmission is not modelled
Breaking the Hard-to-Abate Bottleneck in China's Path to Carbon Neutrality with Clean Hydrogen	X. Yang, Nielsen, Song, and McElroy (2022)	Energy system optimization model	China	The key role of clean hydrogen for the hard-to-abate sectors	Model and analysis specific to China;<br>Cross-border transmission not considered
Renewable Energy Outlook for ASEAN: Towards a Regional Energy Transition (2nd Edition)	IRENA (2022)	Energy systems modelling and projections	ASEAN	ASEAN's economy-wide energy projections (including two net-zero scenarios)	Only technical renewable potentials;<br>Renewable shares in generation are set through a constraint (i.e., 90% and 100%), not results from optimization
The 8th ASEAN Energy Outlook	ACE (2024)	Energy systems modelling and projections	ASEAN	ASEAN's economy-wide energy projections (including a net-zero scenario)	Results only available for the whole ASEAN;<br>Cross-border transmission is not explicitly modelled
An Evaluation of ASEAN Renewable Energy Path to Carbon Neutrality	Fahim, De Silva, Hussain, Shezan, and Yassin (2023)	Policy analysis of climate change mitigation	ASEAN	Policies and actions must be altered to achieve decarbonization	Cross-border transmission and CCS are not considered;<br>No modelling results
Moving beyond the NDCs: ASEAN Pathways to a Net-Zero Emissions Power Sector in 2050	Handayani et al. (2022)	Energy systems modelling and projections	ASEAN	Net-zero pathways for ASEAN's power sector	Cross-border transmission is not modelled;<br>Only scenarios with technical renewable potentials are considered
Optimizing Long-Term Investments for a Sustainable Development of the ASEAN Power System	Huber, Roger, and Hamacher (2015)	Energy system optimization model	ASEAN and Papua New Guinea	The key role of cross-border transmission and renewables in sustainable power systems	Results only available for a single year;<br>Actual climate targets are not incorporated;<br>Only technical renewable potentials are considered
Decarbonization of ASEAN's Power Sector: A Holistic Approach	Lau (2023)	Technology screening and assessment	ASEAN	Country-specific ranking of technologies for decarbonization	Analysis based on the year 2019, no cost-optimal projections through 2050;<br>Cross-border transmission is not considered;

Low-Cost, Low-Emission 100% Renewable Electricity in Southeast Asia Supported by Pumped Hydro Storage	Lu, Blakers, Stocks, and Do (2021)	Energy system optimization model	ASEAN and Timor-Leste	The cost-optimal profiles and storage requirements for a 100% renewable power system	Results only available for 2050;<br>CCS is not considered;<br>Coal- and gas-fired generation are not modelled (aggregated to a single technology)
Integrating 100% Renewable Energy into Electricity Systems: A Net-Zero Analysis for Cambodia, Laos, and Myanmar	Handayani, Overland, Suryadi, and Vakulchuk (2023)	Energy system optimization model	Cambodia, Lao PDR and Myanmar	Cost-optimal generation profiles of a 100% renewable power system for selected ASEAN countries	Results only available for three ASEAN countries;<br>Cross-border transmission and CCS are not modelled
Net-Zero Energy Transition in ASEAN Countries: The Evolutionary Model Brings Novel Perspectives to the Cooperative Mechanism of Climate Governance	Hu and Weng (2024)	Index decomposition of CO2 emissions;<br>Machine learning algorithms	ASEAN	Factors affecting ASEAN's historical emissions, and future emissions pathways	Emissions pathways based on machine learning methods may not be cost-optimal;<br>No results for power sector or investment
Policies toward Net-Zero: Benchmarking the Economic Competitiveness of Nuclear against Wind and Solar Energy	Nian, Mignacca, and Locatelli (2022)	Life Cycle Analysis;<br>Techno-Economic Assessment	Indonesia, Malaysia, Philippines, Thailand, and Vietnam	Nuclear can be economically competitive in the net-zero pathways	Results only for specific ASEAN countries;<br>Cross-border transmission and CCS are not considered;<br>Pathways may not be cost-optimal
Carbon Neutrality Potential of the ASEAN-5 Countries: Implications from Asymmetric Effects of Income Inequality on Renewable Energy Consumption	Tan and Uprasen (2021)	Econometric analysis;<br>Renewable energy consumption	Indonesia, Malaysia, Philippines, Singapore and Thailand	Income inequality can affect carbon neutrality potential through renewable energy consumption	Econometric analysis based on historical data, no projections;<br>Results only available for selected ASEAN countries;<br>Cross-border transmission and CCS are not considered
The Status Quo, Dilemma, and Transformation Path of the Carbon Neutrality-Related Policy of the ASEAN	F. Yang and Li (2024)	Policy analysis of climate change	ASEAN	ASEAN's challenges in achieving net-zero, e.g., unstable policies, and difficulties in regional coordination	Policy assessments based on historical data; no future pathways
Defining a 'Just Energy Investment' for the ASEAN Just Transition	Heffron, Merdekawati, Suryadi, and Yurnaidi (2024)	Composite index;<br>Just energy transition	ASEAN	This study proposes an index of Just Energy Investment, based on five energy justice principles	Analysis on the institutional environment for energy transition;<br>No cost-optimal energy transition pathways

References

ACE. (2019a). *Levelised Costs of Electricity for Renewable Energy Technologies in ASEAN Member States II*. Retrieved from Jakarta: https://aseanenergy.org/levelised-costs-of-electricity-for-renewable-energy-technologies-in-asean-member-states-ii/

ACE. (2019b). *Spatial Estimate of Levelised Costs of Electricity (LCOE) in ASEAN*. Retrieved from Jakarta: https://aseanenergy.org/spatial-estimate-of-levelised-costs-of-electricity-lcoe-in-asean/

ACE. (2020). *The 6th ASEAN Energy Outlook*. Retrieved from Jakarta: https://aseanenergy.org/the-6th-asean-energy-outlook/

ACE. (2024). *The 8th ASEAN Energy Outlook*. Retrieved from Jakarta: https://aseanenergy.org/publications/the-8th-asean-energy-outlook/

Ahmad, S., Ab Kadir, M. Z. A., & Shafie, S. (2011). Current Perspective of the Renewable Energy Development in Malaysia. *Renewable & Sustainable Energy Reviews, 15*(2), 897-904. doi:10.1016/j.rser.2010.11.009

Ahmed, T., Mekhilef, S., Shah, R., & Mithulananthan, N. (2017). Investigation into transmission options for cross-border power trading in ASEAN power grid. *Energy Policy, 108*, 91-101. doi:10.1016/j.enpol.2017.05.020

Albertus, P., Manser, J. S., & Litzelman, S. (2020). Long-Duration Electricity Storage Applications, Economics, and Technologies. *Joule, 4*(1), 21-32. doi:10.1016/j.joule.2019.11.009

Asian Development Bank. (2018a). *Cambodia: Energy Sector Assessment, Strategy, and Road Map*. Manila: Asian Development Bank.

Asian Development Bank. (2018b). *Philippines: Energy Sector Assessment, Strategy, and Road Map*. Manila: Asian Development Bank.

Asian Development Bank. (2019). *Lao People's Democratic Republic: Energy Sector Assessment, Strategy, and Road Map*. Manila: Asian Development Bank.

Baker & McKenzie. (2021). Vietnam: Key Highlights of New Draft of National Power Development Plan (Draft PDP8). Retrieved from https://www.globalcompliancenews.com/2021/03/13/vietnam-key-highlights-of-new-draft-of-national-power-development-plan-draft-pdp8-04032021-2/

Bistline, J. E. T., & Young, D. T. (2022). The Role of Natural Gas in Reaching Net-Zero Emissions in the Electric Sector. *Nature Communications, 13*(1), 4743. doi:10.1038/s41467-022-32468-w

BP. (2021). Statistical Review of World Energy. Retrieved from https://www.bp.com/content/dam/bp/business-sites/en/global/corporate/pdfs/energy-economics/statistical-review/bp-stats-review-2021-full-report.pdf

Chang, Y., Lee, J., Ang, X. W., & Chua, Y. J. (2019). Energy Market Integration in ASEAN: Locational Marginal Pricing and Welfare Implications. *Journal of Asian Economic Integration, 1*(1), 48-72. doi:10.1177/2631684618821568

Davis, S. J., Lewis, N. S., Shaner, M., Aggarwal, S., Arent, D., Azevedo, I. L., . . . Caldeira, K. (2018). Net-Zero Emissions Energy Systems. *Science, 360*(6396), 1419-+. doi:10.1126/science.aas9793

DeAngelo, J., Azevedo, I., Bistline, J., Clarke, L., Luderer, G., Byers, E., & Davis, S. J. (2021). Energy Systems in Scenarios at Net-Zero CO2 Emissions. *Nature Communications, 12*(1), 10. doi:10.1038/s41467-021-26356-y

DOE of the Philippines. (2021). *Philippines Energy Plan 2020-2040*. Manila: Department of Energy (DOE).

EIA. (2023). Annual Energy Outlook 2023. Retrieved from https://www.eia.gov/outlooks/aeo/

EMA. (2021). Singapore Energy Statistics. Retrieved from https://www.ema.gov.sg/Singapore_Energy_Statistics.aspx

EMA. (2022). Charting the Energy Transition to 2050: Energy 2050 Committee Report. Retrieved from https://www.ema.gov.sg/energy-2050-committee-report.aspx

Enerdata. (2021). Power Plant Tracker. Retrieved from https://www.enerdata.net/research/power-plant-database.html

ERIA. (2014). *Investing in Power Grid Interconnection in East Asia*. Retrieved from Jakarta: https://www.eria.org/research/investing-in-power-grid-interconnection-in-east-asia/

ERIA. (2021). *Energy Outlook and Energy Saving Potential in East Asia 2020*. Retrieved from Jakarta: https://www.eria.org/publications/energy-outlook-and-energy-saving-potential-in-east-asia-2020/

European Commission. (2022). PVGIS Photovoltaic Geographical Information System. Retrieved from https://re.jrc.ec.europa.eu/pvg_tools/en/

Fahim, K. E., De Silva, L. C., Hussain, F., Shezan, S. A., & Yassin, H. (2023). An Evaluation of ASEAN Renewable Energy Path to Carbon Neutrality. *Sustainability, 15*(8), 27. doi:10.3390/su15086961

Feenstra, R. C., Markusen, J. R., & Rose, A. K. (2001). Using the Gravity Equation to Differentiate among Alternative Theories of Trade. *Canadian Journal of Economics-Revue Canadienne D Economique, 34*(2), 430-447. doi:10.1111/0008-4085.00082

Goh, T., Ang, B. W., Su, B., & Wang, H. (2018). Drivers of Stagnating Global Carbon Intensity of Electricity and the Way Forward. *Energy Policy, 113*, 149-156. doi:10.1016/j.enpol.2017.10.058
Government of Brunei Darussalam. (2020). Brunei Darussalam Nationally Determined Contribution (NDC) 2020. Retrieved from https://www4.unfccc.int/sites/ndcstaging/PublishedDocuments/Brunei%20Darussalam%20First/Brunei%20Darussalam%27s%20NDC%202020.pdf
Government of Indonesia. (2018). Indonesia Second Biennial Update Report. Retrieved from https://unfccc.int/documents/192165
Government of Indonesia. (2021). Updated Nationally Determined Contribution. Retrieved from https://www4.unfccc.int/sites/ndcstaging/PublishedDocuments/Indonesia%20First/Updated%20NDC%20Indonesia%202021%20-%20corrected%20version.pdf
Government of Myanmar. (2021). Nationally Determined Contributions. Retrieved from https://www4.unfccc.int/sites/ndcstaging/PublishedDocuments/Myanmar%20First/Myanmar%20Updated%20%20NDC%20July%202021.pdf
Government of Singapore. (2020). Singapore's Update of its First Nationally Determined Contribution (NDC) and Accompanying Information. Retrieved from https://www4.unfccc.int/sites/ndcstaging/PublishedDocuments/Singapore%20First/Singapore%27s%20Update%20of%201st%20NDC.pdf
Government of Thailand. (2020). Thailand's Updated Nationally Determined Contribution. Retrieved from https://www4.unfccc.int/sites/ndcstaging/PublishedDocuments/Thailand%20First/Thailand%20Updated%20NDC.pdf
Government of Viet Nam. (2020). Updated Nationally Determined Contribution (NDC). Retrieved from https://www4.unfccc.int/sites/ndcstaging/PublishedDocuments/Viet%20Nam%20First/Viet%20Nam_NDC_2020_Eng.pdf
Handayani, K., Anugrah, P., Goembira, F., Overland, I., Suryadi, B., & Swandaru, A. (2022). Moving beyond the NDCs: ASEAN Pathways to a Net-Zero Emissions Power Sector in 2050. *Applied Energy, 311*, 118580. doi:https://doi.org/10.1016/j.apenergy.2022.118580
Handayani, K., Overland, I., Suryadi, B., & Vakulchuk, R. (2023). Integrating 100% Renewable Energy into Electricity Systems: A Net-Zero Analysis for Cambodia, Laos, and Myanmar. *Energy Reports, 10*, 4849-4869. doi:10.1016/j.egyr.2023.11.005
Heffron, R. J., Merdekawati, M., Suryadi, B., & Yurnaidi, Z. (2024). Defining a 'Just Energy Investment' for the ASEAN Just Transition. *Environmental and Sustainability Indicators, 22*, 11. doi:10.1016/j.indic.2024.100367
Howells, M., Rogner, H., Strachan, N., Heaps, C., Huntington, H., Kypreos, S., . . . Roehrl, A. (2011). OSeMOSYS: The Open Source Energy Modeling System: An introduction to its ethos, structure and development. *Energy Policy, 39*(10), 5850-5870. doi:10.1016/j.enpol.2011.06.033
Hu, Y., & Weng, L. F. (2024). Net-Zero Energy Transition in ASEAN Countries: The Evolutionary Model Brings Novel Perspectives to the Cooperative Mechanism of Climate Governance. *Journal of Environmental Management, 351*, 10. doi:10.1016/j.jenvman.2023.119999
Huber, M., Roger, A., & Hamacher, T. (2015). Optimizing Long-Term Investments for a Sustainable Development of the ASEAN Power System. *Energy, 88*, 180-193. doi:10.1016/j.energy.2015.04.065
IEA. (2019a). *Establishing multilateral power trade in ASEAN*. Retrieved from Paris: https://www.iea.org/reports/establishing-multilateral-power-trade-in-asean
IEA. (2019b). *Southeast Asia Energy Outlook 2019*. Retrieved from Paris: https://www.iea.org/reports/southeast-asia-energy-outlook-2019
IEA. (2020a). *CCUS in Clean Energy Transitions*. Retrieved from Paris: https://www.iea.org/reports/ccus-in-clean-energy-transitions

IEA. (2020b). *Electricity Market Report*. Retrieved from Paris: https://www.iea.org/reports/electricity-market-report-december-2020
IEA. (2020c). *Projected Costs of Generating Electricity 2020*. Retrieved from Paris: https://www.iea.org/reports/projected-costs-of-generating-electricity-2020
IEA. (2021a). CO2 Emissions from Fuel Combustion Statistics. Retrieved from https://www-oecd-ilibrary-org.libproxy1.nus.edu.sg/energy/data/iea-co2-emissions-from-fuel-combustion-statistics_co2-data-en
IEA. (2021b). Energy Prices. Retrieved from https://www.iea.org/data-and-statistics/data-product/energy-prices
IEA. (2021c). IEA World Energy Statistics and Balances. Retrieved from https://www.oecd-ilibrary.org/energy/data/iea-world-energy-statistics-and-balances_enestats-data-en
IEA. (2022a). *Global Energy and Climate Model*. Retrieved from Paris: https://www.iea.org/reports/global-energy-and-climate-model
IEA. (2022b). *Southeast Asia Energy Outlook 2022*. Retrieved from Paris: https://www.iea.org/reports/southeast-asia-energy-outlook-2022
IEA. (2023). *World Energy Outlook 2023*. Retrieved from Paris: https://www.iea.org/reports/world-energy-outlook-2023
IRENA. (2012). *Renewable Energy Cost Analysis - Biomass for Power Generation*. Abu Dhabi: IRENA.
IRENA. (2017a). *Renewable Energy Outlook: Thailand*. Abu Dhabi: IRENA.
IRENA. (2017b). *Renewables Readiness Assessment: The Philippines*. Abu Dhabi: IRENA.
IRENA. (2021a). *Renewable Capacity Statistics 2021*. Abu Dhabi: IRENA.
IRENA. (2021b). Statistical Profiles. Retrieved from https://www.irena.org/Statistics/Statistical-Profiles
IRENA. (2022). *Renewable Energy Outlook for ASEAN: Towards a Regional Energy Transition (2nd Edition)*. Retrieved from Abu Dhabi: https://www.irena.org/publications/2022/Sep/Renewable-Energy-Outlook-for-ASEAN-2nd-edition
Jenkins, J. D., Mayfield, E. N., Larson, E. D., Pacala, S. W., & Greig, C. (2021). Mission Net-Zero America: The Nation-Building Path to a Prosperous, Net-Zero Emissions Economy. *Joule, 5*(11), 2755-2761. doi:10.1016/j.joule.2021.10.016
Kingdom of Cambodia. (2020). Cambodia's Updated Nationally Determined Contribution. Retrieved from https://www4.unfccc.int/sites/ndcstaging/PublishedDocuments/Cambodia%20First/20201231_NDC_Update_Cambodia.pdf
Land Transport Authority. (2022). Electric Vehicles. Retrieved from https://www.lta.gov.sg/content/ltagov/en/industry_innovations/technologies/electric_vehicles.html
Lau, H. C. (2023). Decarbonization of ASEAN's Power Sector: A Holistic Approach. *Energy Reports, 9*, 676-702. doi:10.1016/j.egyr.2022.11.209
Lee, N., Grue, N., & Rosenlieb, E. (2018). Task 2 Report – A GIS-Based Technical Potential Assessment of Domestic Energy Resources for Electricity Generation. Retrieved from https://www.nrel.gov/usaid-partnership/identifying-renewable-energy-opportunities-lao.html
Lu, B., Blakers, A., Stocks, M., & Do, T. N. (2021). Low-Cost, Low-Emission 100% Renewable Electricity in Southeast Asia Supported by Pumped Hydro Storage. *Energy, 236*, 15. doi:10.1016/j.energy.2021.121387
Lugovoy, O., Gao, S., Gao, J., & Jiang, K. (2021). Feasibility Study of China's Electric Power Sector Transition to Zero Emissions by 2050. *Energy Economics, 96*, 105176. doi:https://doi.org/10.1016/j.eneco.2021.105176
Meinshausen, M., Lewis, J., McGlade, C., Gütschow, J., Nicholls, Z., Burdon, R., . . . Hackmann, B. (2022). Realization of Paris Agreement Pledges may Limit Warming just below 2 °C. *Nature, 604*(7905), 304-+. doi:10.1038/s41586-022-04553-z
MEMR, Danish Energy Agency, & Ea Energy Analysis. (2021). *Technology Data for the Indonesian Power Sector-Catalogue for Generation and Storage of Electricity*. Retrieved

from https://ens.dk/sites/ens.dk/files/Globalcooperation/technology_data_for_the_indonesian_power_sector_-_final.pdf
National Energy Council of Indonesia. (2019). Indonesia Energy Outlook 2019. Retrieved from https://www.esdm.go.id/assets/media/content/content-indonesia-energy-outlook-2019-english-version.pdf
NCCS. (2020). *Update of the Solar Photovoltaic (PV) Roadmap for Singapore*. Retrieved from Singapore: https://www.nccs.gov.sg/docs/default-source/default-document-library/Solar%20PV%20Roadmap%20for%20Singapore%202020.pdf
NCCS. (2021). Singapore's Long-Term Low-Emissions Development Strategy. Retrieved from https://www.nccs.gov.sg/media/publications/singapores-long-term-low-emissions-development-strategy
Nian, V. C., Mignacca, B., & Locatelli, G. (2022). Policies toward Net-Zero: Benchmarking the Economic Competitiveness of Nuclear against Wind and Solar Energy. *Applied Energy, 320*, 15. doi:10.1016/j.apenergy.2022.119275
NREL. (2019). Annual Technology Baseline: Electricity. Retrieved from https://atb-archive.nrel.gov/electricity/2019/index.html?t=cg
NREL. (2021). Annual Technology Baseline. Retrieved from https://atb.nrel.gov/
NREL. (2022). RE Explorer. Retrieved from https://www.re-explorer.org/index.html
OPIS. (2023). McCloskey by OPIS. Retrieved from https://www.opisnet.com/commodities/coal-metals-mining/
Ou, Y., Roney, C., Alsalam, J., Calvin, K., Creason, J., Edmonds, J., . . . McJeon, H. (2021). Deep Mitigation of CO2 and Non-CO2 Greenhouse Gases toward 1.5 °C and 2 °C Futures. *Nature Communications, 12*(1), 9. doi:10.1038/s41467-021-26509-z
Paardekooper, S., Lund, R. S., Mathiesen, B. V., Chang, M., Petersen, U. R., Grundahl, L., . . . Persson, U. (2018). EU28 Fuel Prices for 2015, 2030 and 2050. In *Heat Roadmap Europe 4: Quantifying the Impact of Low-Carbon Heating and Cooling Roadmaps.*
Paltsev, S., Mehling, M., Winchester, N., Morris, J., & Ledvina, K. (2018). *Pathways to Paris: ASEAN*. Retrieved from Boston: https://globalchange.mit.edu/publication/p2p-asean
Sanchez, D. L., & Kammen, D. M. (2016). A Commercialization Strategy for Carbon-Negative Energy. *Nature Energy, 1*(1), 15002. doi:10.1038/nenergy.2015.2
Sanchez, D. L., Nelson, J. H., Johnston, J., Mileva, A., & Kammen, D. M. (2015). Biomass Enables the Transition to a Carbon-Negative Power System across Western North America. *Nature Climate Change, 5*(3), 230-234. doi:10.1038/nclimate2488
Siala, K., Chowdhury, A. K., Dang, T. D., & Galelli, S. (2021). Solar Energy and Regional Coordination as a Feasible Alternative to Large Hydropower in Southeast Asia. *Nature Communications, 12*(1), 4159. doi:10.1038/s41467-021-24437-6
Siala, K., de la Rúa, C., Lechón, Y., & Hamacher, T. (2019). Towards a sustainable European energy system: Linking optimization models with multi-regional input-output analysis. *Energy Strategy Reviews, 26*, 100391. doi:10.1016/j.esr.2019.100391
Stich, J., & Massier, T. (2015). Enhancing the Integration of Renewables by Trans-Border Electricity Trade in ASEAN. In *2015 IEEE Pes Asia-Pacific Power and Energy Engineering Conference*. New York: IEEE.
Tan, Y., & Uprasen, U. (2021). Carbon Neutrality Potential of the ASEAN-5 Countries: Implications from Asymmetric Effects of Income Inequality on Renewable Energy Consumption. *Journal of Environmental Management, 299*, 14. doi:10.1016/j.jenvman.2021.113635
Tsiropoulos, I., Tarvydas, D., & Zucker, A. (2018). *Cost Development of Low Carbon Energy Technologies: Scenario-based Cost Trajectories to 2050: Scenario-based Cost Trajectories to 2050, 2017 Edition*. Luxembourg: Publications Office of the European Union.
Tun, M. M., Juchelkova, D., Win, M. M., Thu, A. M., & Puchor, T. (2019). Biomass Energy: An Overview of Biomass Sources, Energy Potential, and Management in Southeast Asian Countries. *Resources, 8*(2). doi:10.3390/resources8020081

UNFCCC. (2021). End of Coal in Sight at COP26. Retrieved from https://unfccc.int/news/end-of-coal-in-sight-at-cop26

van Soest, H. L., den Elzen, M. G. J., & van Vuuren, D. P. (2021). Net-Zero Emission Targets for Major Emitting Countries Consistent with the Paris Agreement. *Nature Communications, 12*(1), 9. doi:10.1038/s41467-021-22294-x

Vietnam Electricity. (2020). Import of Electricity: Solution to Ensure National Energy Security. Retrieved from https://en.evn.com.vn/d6/news/Import-of-electricity-Solution-to-ensure-national-energy-security-66-163-1834.aspx

Wang, H., Su, B., Mu, H., Li, N., Gui, S., Duan, Y., . . . Kong, X. (2020). Optimal way to achieve renewable portfolio standard policy goals from the electricity generation, transmission, and trading perspectives in southern China. *Energy Policy, 139*, 111319. doi:10.1016/j.enpol.2020.111319

Wang, Y., Wang, R., Tanaka, K., Ciais, P., Penuelas, J., Balkanski, Y., . . . Zhang, R. (2023). Accelerating the Energy Transition towards Photovoltaic and Wind in China. *Nature, 619*(7971), 761-767. doi:10.1038/s41586-023-06180-8

Watcharejyothin, M., & Shrestha, R. M. (2009). Regional energy resource development and energy security under CO2 emission constraint in the greater Mekong sub-region countries (GMS). *Energy Policy, 37*(11), 4428-4441. doi:10.1016/j.enpol.2009.05.063

Weng, Y. W., Cai, W. J., & Wang, C. (2021). Evaluating the Use of BECCS and Afforestation under China's Carbon-Neutral Target for 2060. *Applied Energy, 299*, 13. doi:10.1016/j.apenergy.2021.117263

Williams, J. H., Jones, R. A., Haley, B., Kwok, G., Hargreaves, J., Farbes, J., & Torn, M. S. (2021). Carbon-Neutral Pathways for the United States. *Agu Advances, 2*(1), 25. doi:10.1029/2020av000284

Wolfram, P., Kyle, P., Fuhrman, J., O'Rourke, P., & McJeon, H. (2024). The Hydrogen Economy Can Reduce Costs of Climate Change Mitigation by up to 22%. *One Earth, 7*(5), 12. doi:10.1016/j.oneear.2024.04.012

World Bank. (2021). Global Solar Atlas. Retrieved from https://globalsolaratlas.info/global-pv-potential-study

World Bank. (2023). Commodity Markets. Retrieved from https://www.worldbank.org/en/research/commodity-markets

Yang, F., & Li, C. T. (2024). The Status Quo, Dilemma, and Transformation Path of the Carbon Neutrality-Related Policy of the ASEAN. *Sustainability, 16*(3), 25. doi:10.3390/su16031348

Yang, X., Nielsen, C. P., Song, S. J., & McElroy, M. B. (2022). Breaking the Hard-to-Abate Bottleneck in China's Path to Carbon Neutrality with Clean Hydrogen. *Nature Energy, 7*(10), 955-965. doi:10.1038/s41560-022-01114-6

Yu, Z., Bowen, B. H., Sparrow, F. T., Siriariyaporn, V., & Yu, L. (2005). Integrated Energy Resources Planning for the ASEAN Countries and Southern China. *Oil, Gas & Energy Law Journal*, 13.
